# Complete Removal of Extracellular IgG Antibodies in a Randomized Dose-Escalation Phase I Study with the Bacterial Enzyme IdeS – A Novel Therapeutic Opportunity

**DOI:** 10.1371/journal.pone.0132011

**Published:** 2015-07-15

**Authors:** Lena Winstedt, Sofia Järnum, Emma Andersson Nordahl, Andreas Olsson, Anna Runström, Robert Bockermann, Christofer Karlsson, Johan Malmström, Gabriella Samuelsson Palmgren, Ulf Malmqvist, Lars Björck, Christian Kjellman

**Affiliations:** 1 Hansa Medical AB, Scheelevägen 22, P.O. Box 785, SE-22007 Lund, Sweden; 2 Department of Clinical Sciences, Lund, Lund University, SE-221 84 Lund, Sweden; 3 Clinical Trials Unit, Skåne University Hospital, Lund, Sweden; National Cancer Institute, UNITED STATES

## Abstract

IdeS is a streptococcal protease that cleaves IgG antibodies into F(ab’)_2_ and Fc fragments with a unique degree of specificity, thereby providing a novel treatment opportunity of IgG-driven autoimmune conditions and antibody mediated transplant rejection. Here we report the results from a first in man, double blinded and randomized study with single ascending doses of IdeS in healthy, male subjects. Twenty healthy subjects were given intravenous single ascending doses of IdeS. With impressive efficacy IdeS cleaved the entire plasma IgG-pool only minutes after dosing. IgG reached nadir 6-24 hours after dosing and then slowly recovered. The half-life of IdeS was 4.9 (±2.8) hours at 0.24 mg/kg with the main fraction eliminated during 24 hours. Already two hours after IdeS-dosing, the phagocytic capacity of IgG/IgG-fragments was reduced to background levels. Importantly, IdeS has the capacity to inactivate Fc-mediated effector function *in vivo*, was considered safe with no serious adverse events, and without dose limiting toxicity in this study. The complete, rapid, but temporary removal of IgG provides a new potent therapeutic opportunity in IgG-mediated pathogenic conditions.

**Trial Registration:**

ClinicalTrials.gov NCT01802697

## Introduction

In 2001, a cysteine protease was discovered and purified [1] from *Streptococcus pyogenes*, a significant human pathogen often referred to in popular press as the ‘killer bug’. It is responsible for over 0.5 million deaths and at least 700 million cases of pharyngitis and skin infections annually [2]. The protease, IdeS (Immunoglobulin G-degrading enzyme of *S* *treptococcus pyogenes*), has a unique degree of specificity; Immunoglobulin G (IgG) is its only substrate. IdeS is a *S*. *pyogenes* virulence factor by removing the Fc region from host IgG bound to the streptococcal surface, thereby protecting the bacteria against phagocytic killing. The enzyme cleaves IgG in the hinge region, generating one F(ab’)_2_ fragment and one non-covalently linked homo-dimeric Fc fragment. To initiate cleavage IdeS has to bind to the Fc part of IgG, and the remarkable specificity for IgG is explained by the requirement for this initial protein-protein interaction [3,4].

Autoimmune diseases represent an enormous medical problem affecting approximately five percent of the human population (NIH Autoimmune Coordinating Committee 2002). Pathogenic IgG are recognized in many autoimmune conditions as well as in acute transplant rejections. The properties of IdeS raised the question whether the enzyme could be utilized as a drug to disarm pathogenic IgG. A series of studies with IdeS demonstrated rapid and effective cleavage of the entire IgG pool in human blood *in vitro*, complete but temporary IgG removal in rabbits, and curing of mice with various IgG-driven autoimmune diseases [5–9].

IdeS efficiently cleaves rabbit IgG and the four subclasses of human IgG [1,10,11]. IdeS was demonstrated to efficiently abrogate disease induction in collagen antibody induced arthritis (CAIA) if administered within 24 hours after onset. Disease was induced in mice with monoclonal antibodies against type II collagen that were sensitive to IdeS cleavage (i.e. IgG2a^b^) [6]. IdeS did not block ongoing severe arthritis indicating that the later destruction of cartilage was not primarily driven by collagen specific IgG. IdeS was also demonstrated to prevent depletion of platelets and petechial hemorrhaging in a mouse model of immune thrombocytopenia (ITP) induced with a rabbit anti-platelet polyclonal [5]. IdeS was effective also when treatment was initiated at low platelet count. In a mouse model of anti-glomerular basement membrane (GBM) disease. IdeS was demonstrated to prevent albuminuria, deposition of C1q and C3 in glomeruli and to diminish leukocyte infiltration into glomeruli [9]. In this model it was demonstrated that IdeS could neutralize IgG already bound to the GBM. The effect of IdeS in these previous animal studies was dramatic, and provided a basis for the present investigation where healthy human subjects received ascending doses of intravenous IdeS. The primary objectives in this present study were to assess the safety and tolerability of IdeS following intravenous administration of single ascending doses in healthy male subjects. Additionally, secondary objectives were to determine the pharmacokinektic (PK), the immunogenicity profile and the pharmacodynamics (PD) profile of IdeS, which is determined by assessing the efficacy of IdeS in cleaving IgG. The results uncover a new therapeutic concept to eliminate pathogenic IgG.

## Materials and Methods

### Study outline

**Fig 1:**
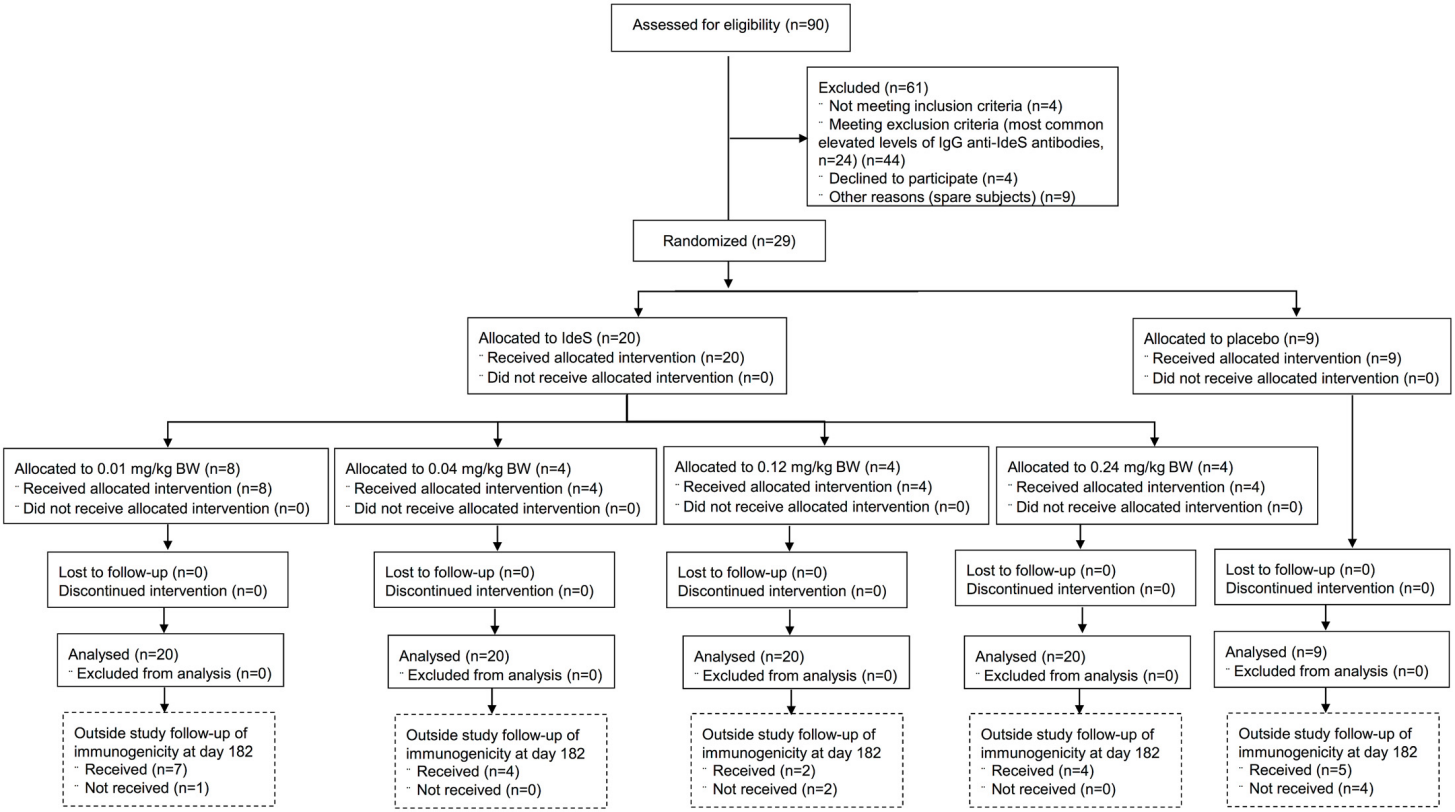
Participant flow diagram for the safety study on IdeS in healthy volunteers. The study ended at day 64, but all subjects were asked to come back for additional sampling at day 182 in order to monitor immunogenicity.

This was a phase I, first in man, double blinded and randomized study with single ascending doses of IdeS in healthy, male subjects (ClinicalTrials.gov Identifier: NCT01802697) conducted in the Clinical Trials Unit at Skåne University Hospital, Lund, Sweden (Fig 1). The protocol was approved by the local ethics committee in Lund (Dnr 2012/782, Etikprövningsnämnden, Östra Vallgatan 14, SE-223 61 Lund, Sweden) prior to recruitment and all subjects signed written informed consent before undergoing any study-specific procedures. The primary objective was to assess the safety and tolerability of IdeS following *i*.*v*. administration of single ascending doses. Secondary objectives were evaluation of IdeS efficacy (i.e. reduction in serum IgG), pharmacokinetics, and immunogenicity in healthy human subjects. Due to the exploratory nature of the study the sample size was based on experience from previous similar phase I studies with other compounds to obtain adequate safety, tolerability and PK data to achieve the objectives of the study whilst exposing as few subjects as possible to study medication and procedures.

The hospital pharmacy was supplied with a randomization list produced by the CRO. The list specified the treatment (IdeS or placebo) per subject number. The allocation within groups was two placebo and four IdeS. The diluted infusion solution of the GMP-produced IdeS (Hansa Medical AB, Sweden) was prepared in a phosphate buffered isotonic salt solution by the hospital pharmacy in an infusion syringe with an infusion set including a 0.2 μm filter (B. Braun, Germany). The syringes were marked by subject number and delivered to the Clinical Trial Unit. The selected starting dose was 10-times below the pre-clinically determined Minimal Anticipated Biological Effect Level (MABEL) and 200-times below the No Observed Adverse Effect Level (NOAEL) determined during animal toxicology. The study design allowed gradual escalation of the dose with intensive safety monitoring. The starting dose was 0.01 mg/kg body weight (BW) (n_IdeS_ = 8 and n_Placebo_ = 4) and after evaluation by a data monitoring committee the dose was stepwise increased to 0.04 mg/kg BW (n_IdeS_ = 4 and n_Placebo_ = 2), 0.12 mg/kg BW (n_IdeS_ = 4 and n_Placebo_ = 1) and 0.24 mg/kg BW (n_IdeS_ = 4 and n_Placebo_ = 2). An unfortunate high number of exclusions were made during the recruitment of subjects to the 0.12 mg/kg dose group. The study protocol specified that total pIgG levels were monitored as part of safety and that antibiotics were given to all subjects until pIgG levels had normalized. Thus, the data monitoring committee was presented with safety data for the five subjects randomized in the 0.12 mg/kg group and it was obvious from the pIgG safety data that four subjects had received the active drug and a decision to proceed to the next dose level was made. Due to this decision only five subjects were recruited to the 0.12 mg/kg group (4 active + 1 placebo).

First subject was enrolled in February 2013 and last-subject-last-visit was in December 2013. For inclusion in the study, subjects had to be healthy, aged 18–45 years (actual median: 23, range: 20–41), have suitable veins for cannulation, a body mass index (BMI) between 19 and 30 kg/m^2^ (actual median: 23, range: 20–30) and weigh 50–100 kg (actual median: 76, range: 59–100). Subjects were excluded from the study if they had, or had a history of, any clinically significant immunodeficiency including but not limited to immunoglobulin A deficiency, had elevated levels of anti-IdeS IgG (>15 mg/L), tested positive for serum hepatitis B surface antigen, hepatitis C antibody, human immunodeficiency virus (HIV), ongoing tuberculosis, ongoing syphilis, active herpes simplex or herpes zoster infection during screening.

Screening of subjects was started at the earliest 28 days and the latest five days prior to dosing. Each of the 29 subjects included had a three-day admission period at the Clinical Trials Unit and were randomized to either IdeS or placebo (phosphate buffered saline) and dosed the morning after admission. Two subjects in each dose group were dosed on the first day (one IdeS and one placebo) and the next subjects in the group were dosed after at least one week. After each dose group the data monitoring committee assessed the safety data and decided upon the next dose level. The time from the last dose at one dose level to the initiation of next dose level was at least 14 days. The infusions were given during 30 minutes for the first two subjects in each group and during 15 minutes for subsequent subjects in the group. During the admission period intensive safety monitoring and serial blood samplings for safety, pharmacokinetics, efficacy and anti-drug antibodies were performed. The subjects were discharged on day 4 and conducted at least eight intermediate follow-up visits with medical examination and blood sampling until the end of study at day 64. Furthermore, all subjects were asked to come back for additional serum samples on day 182.

All subjects participating in the study were treated with antibiotics (amoxicillin plus clavulanate or doxycycline if hypersensitive to beta-lactams) as prophylaxis against bacterial infections. Prophylaxis treatment started on the dosing day and continued until plasma IgG levels were >4.5 g/L. No other concomitant medication or therapy was allowed except paracetamol during the first 28 days following dosing unless prescribed by the investigator and considered necessary for the subject’s safety and well-being.

Additional details regarding the study protocol can be found in S1 Protocol and S1 Checklist.

### Safety assessments

Adverse events (AEs) were collected from the time of admission and throughout the study period. Information about AEs included description, start/stop time, grade, severity, causality (unlikely, possible or probable), action taken and outcome. Vital signs, body temperature, heart rate and supine blood pressure were recorded regularly during the admission period and at all subsequent visits. In addition, the subjects were monitored with a 5-lead telemetric ECG during the infusion and the following 24 hours. Safety samples for clinical chemistry, hematology, coagulation, safety biomarkers (IL-6, IL-8 and TNFα) and plasma IgG were analyzed using routine methods at Labmedicin Skåne, Sweden. Urinalysis (U-glucose, U-hemoglobin and U-protein) was assessed using Multistix (Siemens, Germany).

### Serum samples for efficacy, pharmacokinetics and anti-IdeS antibody evaluation

Blood samples intended for efficacy studies were collected in modified CAT serum BD vacutainer tubes (BD Diagnostics, NJ, USA) containing 2 mM of the protease inhibitor iodoacetic acid in order to prevent further proteolytic cleavage by IdeS. Blood sampling was performed at the following time-points for the 0.01 and 0.04 mg/kg BW dose groups: pre-dose and 1 min before end of infusion (14 or 29 min). For the 0.12 and 0.24 mg/kg BW dose groups the following time-points were collected: pre-dose, 1 min before end of infusion (14 or 29 min), 5 min after end of infusion (20 or 35 min) and 45 min after end of infusion (1 h or 1 h and 15 min). In addition samples were collected 2, 6, 24, 48 and 72 h after start of infusion as well as on day 7, 14, 21, 28 and 64 after dosing. Blood samples intended for pharmacokinetic studies were collected in regular serum BD vacutainer tubes at the following time-points for the 0.01 and 0.04 mg/kg BW dose groups: pre-dose and 1 min before end of infusion (14 or 29 min). For the 0.12 and 0.24 mg/kg BW dose groups the following time-points were collected: pre-dose, 1 min before end of infusion, 5 min after end of infusion, 45 min after end of infusion and 2, 6, 24, 48, 72 and 144 h after dosing. Blood samples intended for anti-IdeS antibody analysis were collected in regular serum BD vacutainers at day 1 (pre-dose), 2 (24 h), 3 (48 h), 4 (72 h), 7 (1 wk), 14, (2 wk), 21 (3 wk), 28 (4 wk) and 64 (2 mo) after dosing for all dose groups. Outside the study, all subjects were asked to come back for additional serum samples on day 182 (6 mo). All samples were stored below -60°C until analyzed.

### Efficacy assessment

IdeS cleavage and processing of IgG were investigated with different methods; ELISAs were used to determine IgG and IgG fragments in serum and to investigate the dynamics of the F(ab’)_2_ and Fc containing fragments. The quantitative assays could not completely differentiate between the IdeS cleavage products; i.e. F(ab’)_2_, Fc and single cleaved IgG (scIgG). The ELISA developed and performed by Covance Laboratories Limited (Harrogate, UK) measured intact IgG and scIgG. The Fab ELISA measured all Fab containing IgG fragments; i.e. intact IgG, scIgG and F(ab’)_2_ and the Fc ELISA measured all Fc containing fragments; i.e. intact IgG, scIgG and free Fc. In order to further evaluate the quantitative data the serum samples were also analyzed using qualitative SDS-PAGE.

The assay performed by Covance Laboratories Ltd, UK, was formally validated. Briefly, serum samples were analyzed by an ELISA where IgG was allowed to bind to the catcher antibody, a F(ab’)_2_ fragment goat anti-human IgG, F(ab’)_2_ fragment specific (#109-006-097, Jackson ImmunoResearch Labs Inc., PA, USA). Quantified human serum protein calibrator (IgG) (X0908, Dako, Denmark) was used for preparation of standards and quality control samples. Bound IgG was detected by the subsequent addition of peroxidase-conjugated F(ab’)_2_ fragment goat anti-human IgG, Fcγ fragment specific (#109-036-098, Jackson ImmunoResearch Labs Inc.) and a chromogenic substrate (TMB). The lower limit of quantification for IgG was 5 ng/mL in serum. The serum analyses were performed at Covance Laboratories Ltd, UK.

The Fc ELISA used a goat anti-human IgG, Fcγ fragment specific, F(ab’)_2_ fragment (#109-006-098, Jackson ImmunoResearch Labs Inc.) as catcher antibody and a biotin conjugated goat anti-human IgG, Fcγ fragment specific, F(ab’)_2_ fragment (# 109-066-098, Jackson ImmunoResearch Labs Inc.) as detector. In the Fab ELISA an affinity purified mouse anti-human IgG, F(ab’)_2_ fragment specific antibody (#209-005-097, Jackson ImmunoResearch Labs Inc.) was used as catcher antibody and biotinylated CaptureSelect IgG-CH1 (#710.3120.100 BAC B.V., Naarden, Netherlands) as detector. A streptavidin-horseradish peroxidase conjugate (SA-HRP) (#21126 Pierce, Thermo Fisher Scientific, Rockford, IL) was used for secondary detection. Calibrator and quality control (QC)-samples (ULOQ, LLOQ, H-OC, M-QC and L-QC) were prepared from normal human IgG (Octagam). All dilutions were performed in PBS + 0.1% bovine serum albumin (BSA) and the Nunc MaxiSorp flat-bottom 96-well microtiter plates (Nunc A/S, Roskilde, Denmark) were washed with PBS containing 0.05% Tween 20. The serum samples and QC-samples were analyzed in triplicates. TMB One component HRP Microwell substrate (#TMBW-1000-01, BioFx Laboratories Inc., MD, USA) was used as a chromogenic substrate and the enzyme reaction was stopped by the addition of 0.5 M H_2_SO_4_. The absorbance was measured in an ELISA plate reader (Multiscan EX, Thermo Electron Corp.) (Software: Ascent Software v. 2.6) at λ = 450 nm.

The Sodium dodecyl sulfate polyacrylamide gel electrophoresis (SDS-PAGE) analyses were performed according to the manufacturer’s instructions (Bio-Rad Laboratories, CA, USA). Briefly, 0.25 μL of serum was separated on 4–20% Mini-PROTEAN TGX precast gels (Bio-Rad) at 200 V for 40 minutes under non-reduced conditions. SeeBlue MW standard (Life Technologies) and an *in house* prepared mix of human IgG, scIgG, F(ab’)_2_ and Fc were used as markers. The gels were stained with GelCode Blue stain reagent (Pierce, Thermo Fisher Scientific, MA, USA) according to the manufacturer’s instructions and the gels were scanned.

### Pharmacokinetics assessment

Four IdeS derived peptides, i.e. AFPYLSTK, AIYVTDSDSNASIGMK, GGIFDAVFTR and LFEYFK, were assayed in serum samples by a qualified liquid chromatography coupled with tandem mass spectrometry (LC-MS/MS) assay [12]. Samples were prepared for MS analysis as previously described [12]. The selected reaction monitoring (SRM) measurements were performed on a TSQ Vantage triple quadruple mass spectrometer (Thermo Fisher Scientific, MA, USA) equipped with a PicoChip column packed with Reprosil-PUR C18 (New Objective, MA, USA) and an Easy-nLC II system (Thermo Fisher Scientific). The raw data was processed and analyzed with SRM analysis software Skyline [13] with manual validation and inspection of the results. The injection volume was 1 μL corresponding to 12.5 nL serum (i.e. 1 μg total protein). Un-normalized peptide Total Peak Areas from IdeS-spiked serum was used for fitting a linear regression curve (label-free protein quantification). The concentrations of the individual peptides in the unknown human samples were interpolated from the linear regression. QC-samples were run every 4–6 analytical sample as described elsewhere [14].

Serum concentration versus time data was analyzed by non-compartmental analysis (NCA) in Phoenix WinNonlin version 6.3, build 6.2.0.495 (Pharsight, St. Louis, Missouri, USA). As no major deviations (>20%) between nominal and actual sampling times and doses were observed, nominal sampling times and doses were used for the NCA calculations. The LC-MS/MS assay has not been validated and no formal lower limit of quantification (LLOQ) has been defined. Pharmacokinetic parameters were calculated for up to 24 hours post dose for all measurable peptides and all individuals in the last two dose groups. For the first two dose groups, PK-samples were only collected at the 1 min before end of infusion time-point.

### Anti-IdeS IgG assessment

An ImmunoCAP test for quantification of anti-IdeS specific IgG was developed by Thermo Fisher Scientific (Phadia) in Uppsala, Sweden. Initial testing indicated that a 3-logarithmic measuring range was possible using the IdeS-ImmunoCAP and the limit of detection for the IgG IdeS-specific ImmunoCAP assay was shown to be seven times below the suggested low assay cut off (i.e. 2.0 mg/L). Analyses of the clinical samples were performed on a Phadia 250 instrument using the IdeS-ImmunoCAP test with one replicate according to the Phadia 250 user manual. The ImmunoCAP test was intended for research use only.

### Antigen-specific IgG cleavage efficacy

A research grade ELISA assay was developed at Hansa Medical AB in order to address antigen-specific efficacy at the end of the study. The subjects IgG-response against a vaccine included in the Swedish childhood vaccination program was utilized as a surrogate for lack of auto-antigens in the healthy subjects included in the phase I study. Briefly, Pentavac/Pentaxim (Sanofi Pasteur) was diluted 100-times in PBS prior to coating MaxiSorp plates (Nunc) at 4°C. Normal human IgG (Octagam) was utilized as calibrator and a goat anti-human Fc-specific biotin-SP-conjugated F(ab’)_2_ (#109-066-098, Jackson ImmunoResearch Labs Inc.) as detector. Furthermore, SA-HRP (#21126, Pierce) was used and the signals were developed with TMB One component HRP Microwell Substrate (#TMBW-1000-01, BioFX Laboratories Inc.), stopped with 0.5 M H_2_SO_4._ The absorbance was measured in an ELISA plate reader (Multiscan EX, Thermo Electron Corp.) (Software: Ascent Software v. 2.6) at λ = 450 nm.

### Functional *in vitro* assay

A modified phagocytosis assay was developed based on a previously described method [15]. Yellow-green fluorescent (505/515 nm) neutravidin beads (#F8776, Molecular Probes) were coated over night with biotinylated anti-IgG CH1 (CaptureSelect, #710.3120.100 BAC B.V., Naarden, Netherlands) at 0.1 mg/ml. The CaptureSelect reagent is specific for human heavy chain IgG on the CH1 domain i.e. intact IgG, scIgG and F(ab’)_2_ fragments but IgM will not be captured by this protein. Coated beads were washed and mixed with 100x diluted serum from study subjects and incubated at 37°C for 2 hours to allow IgG in serum to bind to the coated beads. A control was prepared by mixing coated beads with dilution buffer (PBS with 0.1% BSA). All samples were prepared in duplicate. After incubation, IgG-loaded beads were washed and mixed with 75 000 THP-1 cells/sample and incubated in a CO_2_-incubator at 37°C for 1.5–3 hours. After incubation samples were fixed in ice-cold 2% phosphate buffered formalin and the fluorescence uptake in THP-1 cells was monitored using an Accuri C6 flow cytometer. Live THP-1 cells were gated using forward and side scatter and the percent of THP-1 cells positive in the FL2 channel i.e. with engulfed fluorescent beads were further monitored. Due to the limited amount of serum available for this exploratory part of the study we only studied subjects in the highest dose group. Additional details regarding gating scheme for flow cytometry can be found in S1 and S2 Figs.

### Statistical analysis

No formal power calculations have been performed. Means, medians, standard deviations, and basic statistical analysis were performed using the GraphPad Prism 6 software (GraphPad Software, CA, USA).

## Results

A phase I, double blind and randomized study with single ascending doses of IdeS was conducted after approval from Swedish regulatory and ethical authorities. The objective was to assess safety, efficacy, pharmacokinetics, and immunogenicity of IdeS in healthy human subjects following intravenous administration. 29 subjects were included into four dose groups and randomized to either IdeS or placebo. The starting dose was 0.01 mg/kg BW and the highest investigated dose was 0.24 mg/kg BW.

### Assessment of safety

77 adverse events (AEs) were observed in 24 of the 29 subjects with 39 AEs (in 14 subjects) classified as related (i.e. possible or probable). Among these 39 AEs, 35 had a common toxicity criteria grade of 1. Four AEs were grade 2, all from one subject who experienced a probable infusion reaction (Table 1). The reaction resolved within 15 minutes after treatment with antihistamine (2 mg clemastine fumarate *i*.*v*.) and corticosteroids (8 mg betamethasone *i*.*v*.). The IdeS infusion was not interrupted. None of the AEs were reported as serious, met any dose limiting toxicity criteria, or lead to withdrawal of the study drug.

**Table 1:** Summary of adverse events reported for each subject.

		Onset of AE						
Dose	Subject	0–24 hours	2–7 days	8–21 days	22–64 days	Related	Action	Outcome
0.01	103	Nasopharyngitis (1)		Fatigue (1), Daydreaming (1), Tinnitus (1)		Possible	None	Resolved
	106	Nasopharyngitis (1)	Epistaxis (1), Chest discomfort (1), Nightmare (1)			Possible	None	Resolved
	206			Headache (1), Fatigue (1), Nausea (1), Dizziness (1)		Possible	None	Resolved
0.04	303	Nausea (1), Flushing (1)				Possible	None	Resolved
	304		Oropharyngeal pain (1), Nasopharyngitis (1)			Possible	None	Resolved
	305		Blister (1), Headache (1)			Possible	None	Resolved
0.12	404		Diarrhoea (1), Abdominal distension (1)			Possible	None	Resolved
0.24	501	Headache (1), Abdominal discomfort (1)	Nasopharyngitis (1)			Possible	None	Resolved
	504			Asthenia (1)		Possible	None	Resolved
	506	*Flushing (2)*, *Sinus tachycardia (1)*, *Chest discomfort (2)*, *Pharyngeal oedema (2)*, *Nasal congestion (1)*	Throat irritation (1)	Oropharyngeal pain (1), Myalgia (2)		*Probable*, Possible	*Tavegyl*, *Betapred*, None	Resolved
Placebo	101	Peripheral coldness (1), Infusion related reaction (1)	Fatigue (1)			Possible	None	Resolved
	104	Dysgeusia (1)	Nasal congestion (1)			Possible	None	Resolved
	201		Herpes simplex (1)			Possible	None	Resolved
	302		Chills (1)			Possible	None	Resolved

All adverse events (AE) sorted by subject and time of onset after initiation of dosing. All AEs resolved and except for the early (0–24 hours) AEs in subject 506 (italicized in table) did not require any actions. The early AEs in subject 506 were judged probable and the subject received Tavegyl (clemastine fumarate) and Betapred (betamethasone). The common toxicity criteria grade of each AE is indicated within brackets () in the table.

The most common AEs were nasopharyngitis, headache and fatigue. Nasopharyngitis was reported for 10 out of 20 subjects on IdeS and for six out of nine subjects on placebo. Seven subjects reported headache at a total of nine occasions (all on IdeS) whereas six subjects (five on IdeS and one on placebo) reported a total of seven incidences of fatigue.

**Fig 2:**
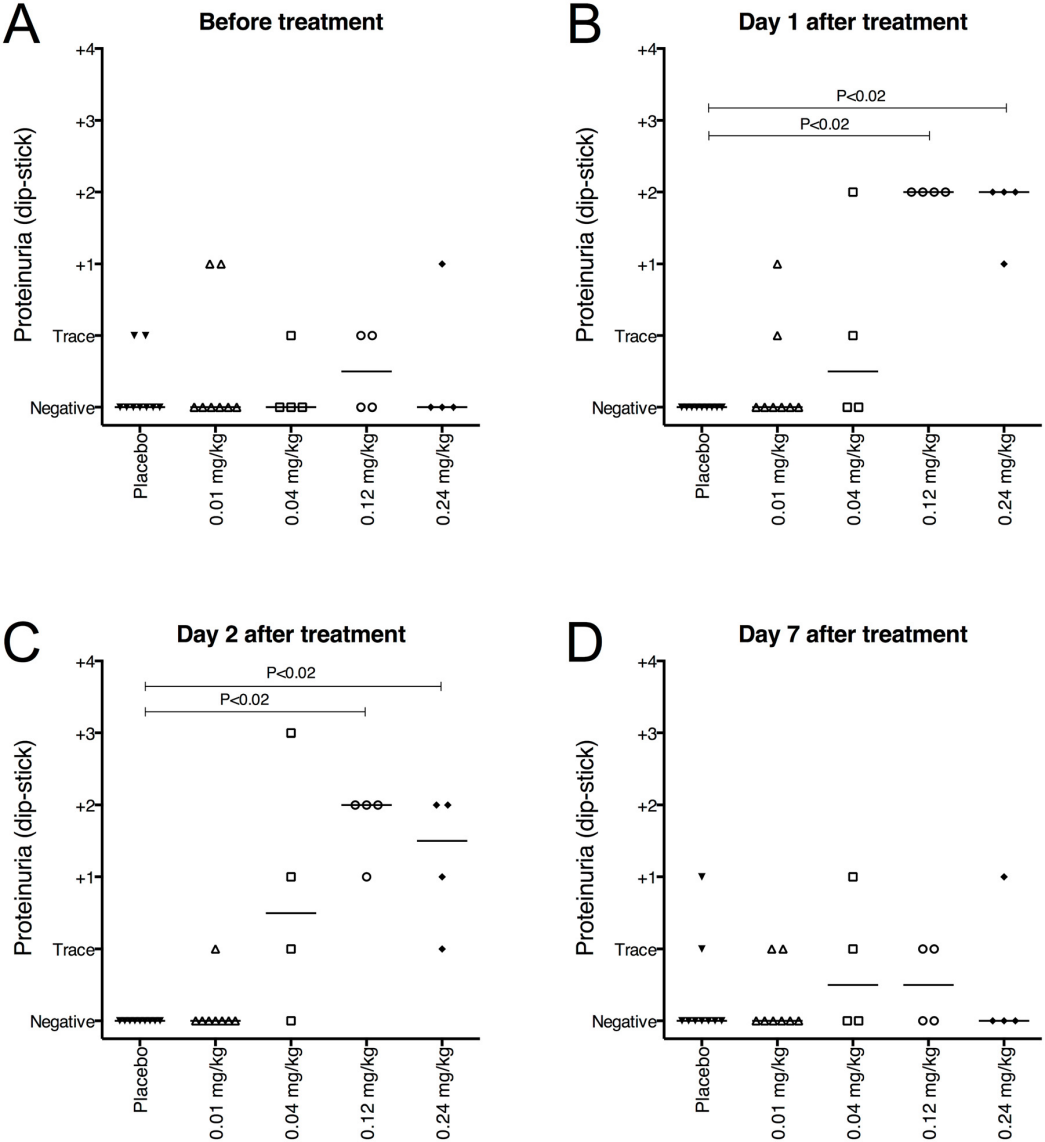
Proteinuria was monitored as a safety assessment throughout the study. Proteinuria was routinely measured at the hospital using Multistix (Siemens) and reported as negative, trace or grade 1–4. Data represents proteinuria in individual subjects at the indicated time-points after treatment. A-C) A mild but significant increase in proteinuria was seen on day 1 (P = 0.0003) and day 2 (P = 0.0002) after treatment with 0.12 and 0.24 mg/kg IdeS. D) On day 7 there were no significant (P = 0.80) differences between groups. The groups (*n*_Placebo_ = 9, *n*_0.01_ = 8, *n*_0.04_ = 4, *n*_0.12_ = 4 and *n*_0.24_ = 4) were compared using Kruskal-Wallis, One-Way ANOVA. The P-values shown in the graph represent comparisons of the mean rank of each dose-group with the placebo group using Dunn’s Multiple Comparison. The placebo group represents a pool of all subjects treated with placebo from all dose-groups.

No clinically significant changes in hematology, clinical chemistry or coagulation were identified. However, a transient proteinuria was observed after 24–48 hours in subjects administered an IdeS dose resulting in significant cleavage of IgG compared to placebo treatment (Fig 2A-2C). This probably reflected the clearance of IgG cleavage products from the circulation since there was a correlation between subjects with elevated proteinuria and subjects where IdeS had an effect. On day 7 there were no significant differences between placebo and IdeS treated groups (Fig 2D). The 0.12 and 0.24 mg/kg BW dose groups showed a statistically significant difference from placebo treated subjects at the 24 and 48 h time-points (P<0.02) (individual results are shown in S1 Raw Data).

### Efficacy and pharmacodynamics of IdeS

**Fig 3:**
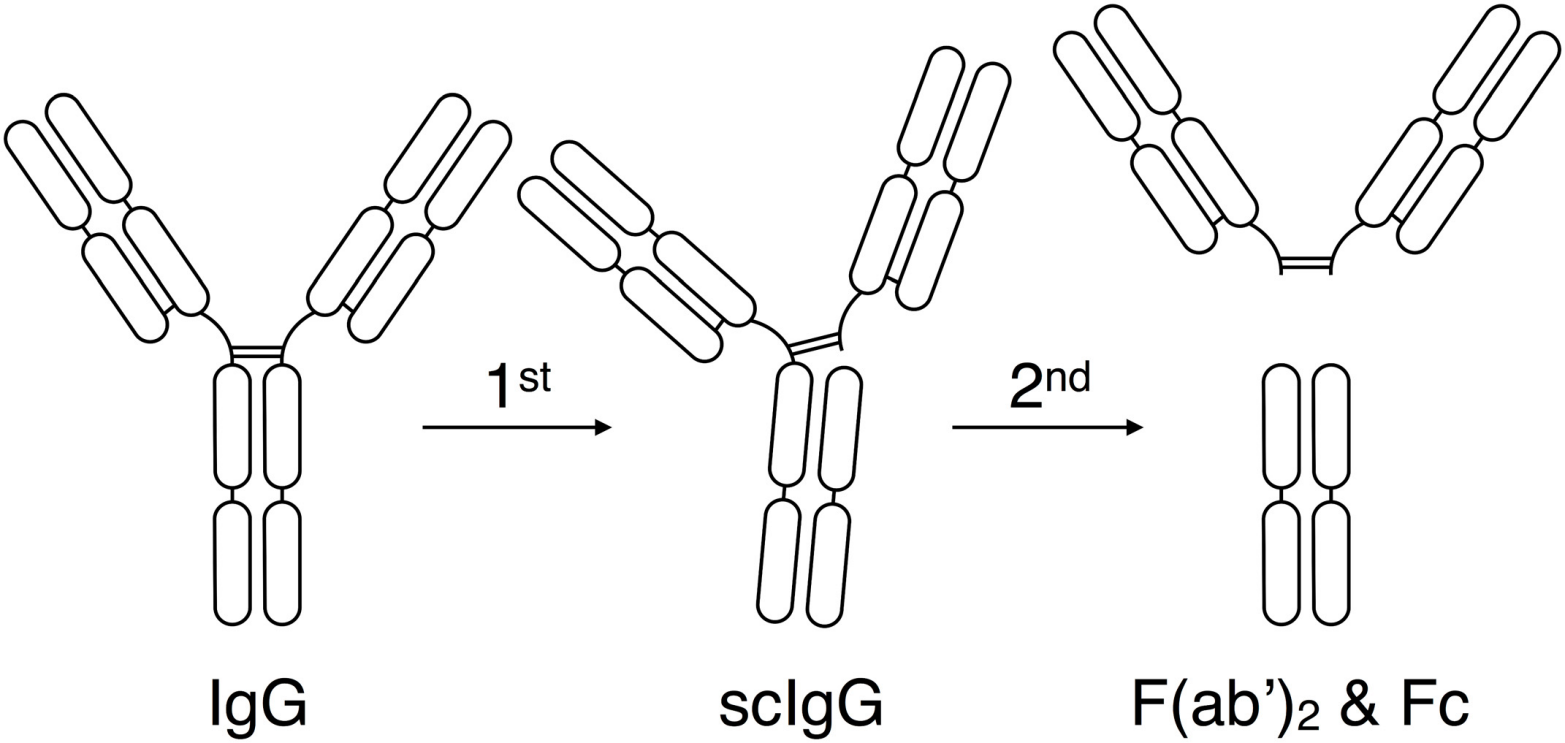
Schematic representation of IgG cleavage by IdeS. Intact human IgG, regardless of isotype, is cleaved by IdeS in two steps. The first step generates a single-cleaved IgG molecule (scIgG) with one intact heavy chain. The second step generates one F(ab’)_2_ fragment and one homo-dimeric Fc fragment held together by non-covalent interactions.

IdeS cleaves IgG in two steps [7,16]. The first reaction is a very rapid and efficient cleavage of one of the two heavy chains generating a single cleaved IgG molecule (scIgG), still having one of the two heavy chains intact. The scIgG is somewhat less sensitive for IdeS activity. The second IdeS cleavage generates one F(ab’)_2_ and one Fc fragment (Fig 3).

**Fig 4:**
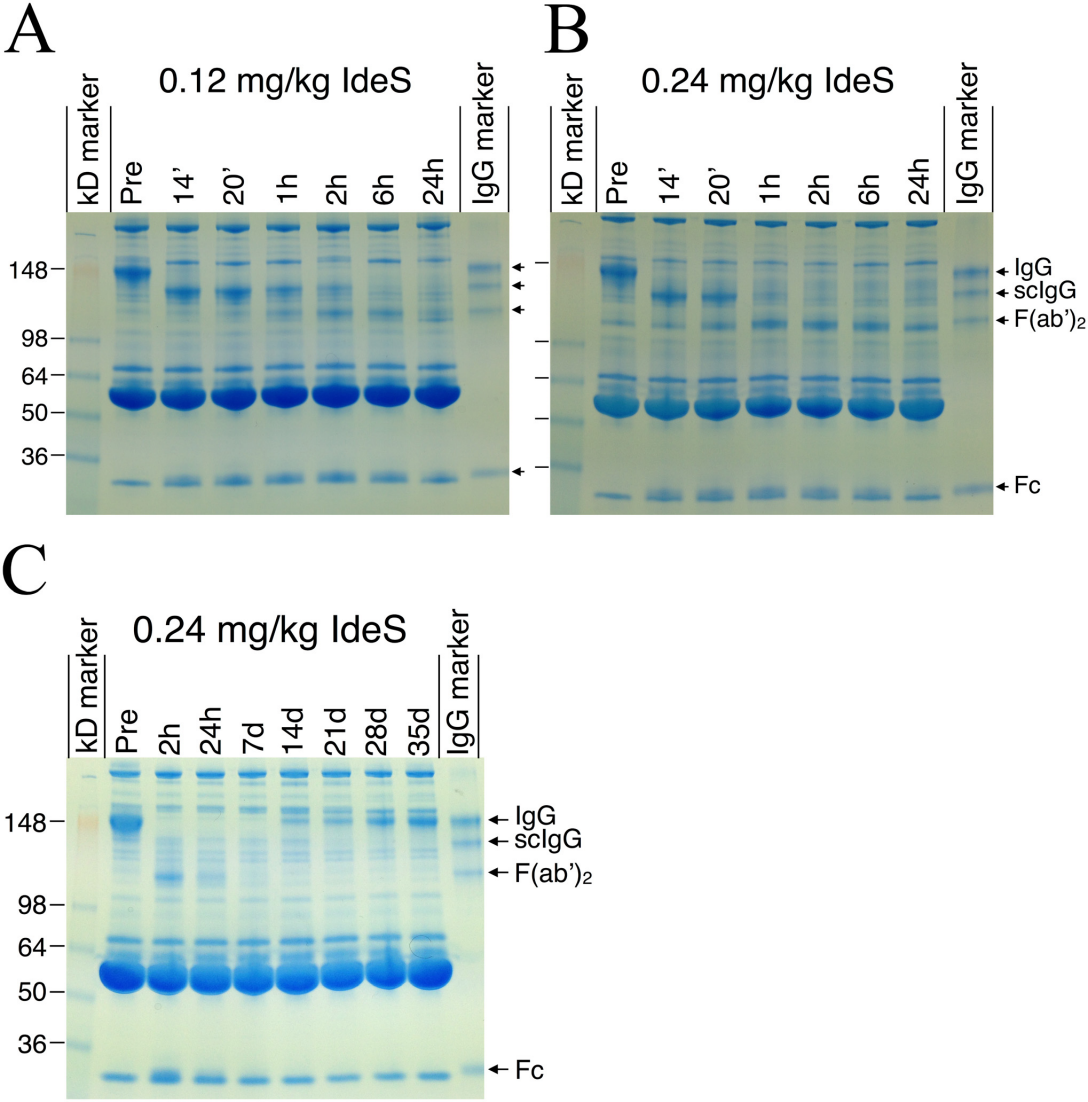
Qualitative pharmacodynamics analysis by SDS-PAGE showed rapid degradation of IgG. Non-reducing SDS-PAGE analysis of serum from subjects dosed with A) 0.12 mg/kg BW IdeS and B) 0.24 mg/kg BW IdeS showing protein banding patterns at pre-dosing, 14 min, 20 min, 1, 2, 6 and 24 hours after dosing. C) IgG recovery in serum from one subject in the 0.24 mg/kg BW group at pre-dosing, 2 hours, 24 hours, 7 days, 14, 21, 28 and 35 days after dosing. Arrows to the right in each figure show the different bands in the IgG-marker containing a mix of human IgG, scIgG, F(ab’)_2_ and Fc. Lines to the left in each figure show the relative molecular mass of the kDa standard. The gels show a representative subject in the 0.12 and 0.24 mg/kg BW IdeS dose groups.

SDS-PAGE analysis under non-reducing conditions revealed that IdeS had close to full effect within six hours in all eight subjects dosed with 0.12 or 0.24 mg/kg BW, i.e. the entire IgG pool was converted into F(ab’)_2_ and Fc fragments (Fig 4A and 4B). The effect was remarkably rapid; the IgG pool was converted into scIgG already during dosing (i.e. one minute prior to full dose) and maximal effect was accomplished 2–6 hours after dosing in all subjects. Newly synthesized intact IgG was clearly detectable in all subjects after one to two weeks, and after three weeks the level of intact IgG constituted the main IgG fraction in serum (Fig 4C).

**Fig 5:**
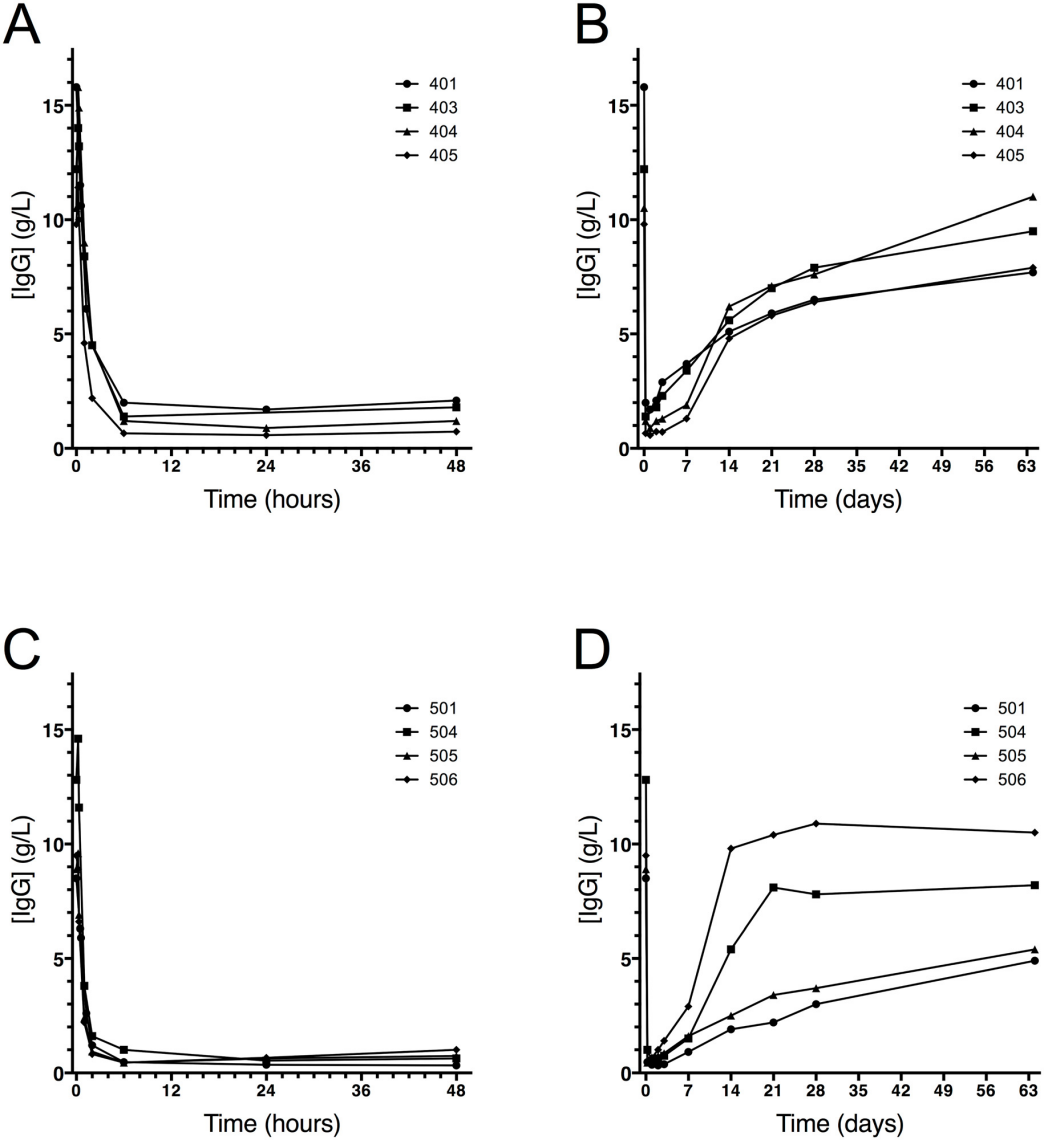
Quantitative pharmacodynamics analysis by ELISA showed rapid degradation of IgG. Serum IgG levels from all four individual subjects dosed with 0.12 mg/kg BW IdeS (A and B) and all four individual subjects dosed with 0.24 mg/kg BW IdeS (C and D) determined using a validated ELISA method performed by Covance Laboratories Ltd, UK. To be able to follow both early, rapid degradation as well as recovery of IgG, graphs A and C show data up to 48 hours after dosing (x-axis in hours) and graphs B and D show data until last visit (x-axis in days).

The rapid cleavage of human IgG into F(ab’)_2_ and Fc was confirmed by ELISA (Fig 5A and 5C), showing that 2–6 hours after dosing low plateau levels reached less than 5% (0.4–0.5 g/L IgG) of the original signal. It was concluded from the SDS-PAGE that the remaining IgG signal mainly originated from scIgG (Fig 4). Using the ELISA, *de novo* production of IgG could be detected two to three days after dosing reaching 0.9–2.9 g/L after one week and 4.9–10.5 g/L after 2 months (Fig 5B and 5D) (individual results are shown in S1 Raw Data). A large inter-individual variation in the rate of IgG recovery was observed and there was no indication of differences between the dose-groups. The F(ab’)_2_ as well as the Fc fragments reached lowest levels between one and seven days post dosing (Fig 4C). The elimination of Fc fragments was somewhat faster than the elimination of F(ab’)_2_ fragments.

### IdeS and antigen-specific IgG antibodies

**Fig 6:**
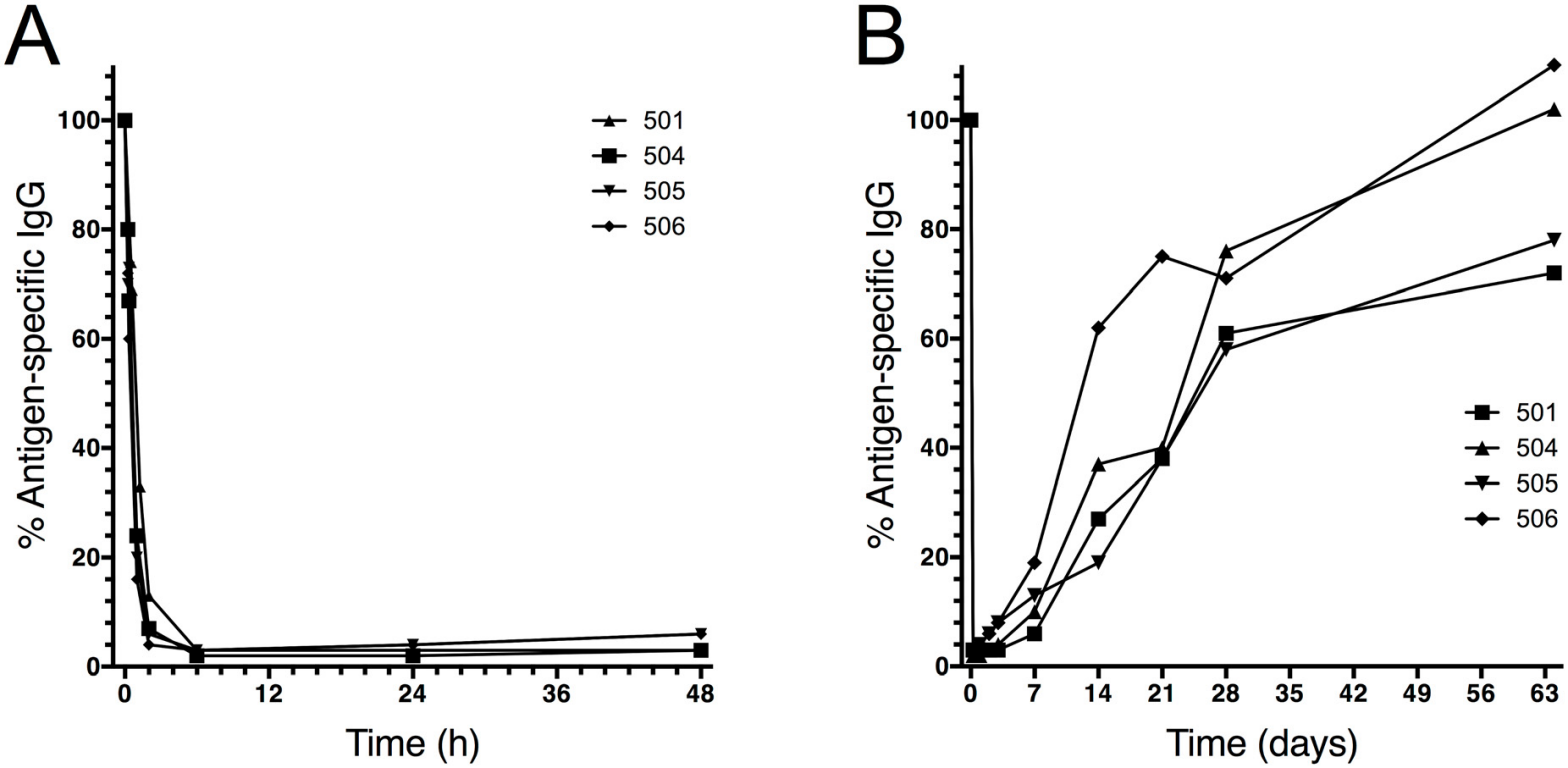
Pharmacodynamics of antigen-specific IgG following IdeS treatment. Human serum samples from the 0.24 mg/kg BW group (*n* = 4) were addressed for presence of specific IgG against a vaccine mixture of antigens (diphtheria, pertussis, tetanus, polio and *Haemophilus influenzae* type b). The results are given as percent remaining IgG on the y-axis compared to the start value for each subject. To be able to follow both early, rapid degradation as well as recovery of IgG, graph A shows data up to 48 hours after dosing (x-axis in hours) and graph B shows data until last visit (x-axis in days).

The vaccine Pentavac is part of the Swedish childhood vaccination program. Consequently, a majority of the Swedish population has IgG antibodies against the antigen components of this vaccine (diphtheria, tetanus, pertussis, polio, and *Haemophilus influenza*e type b). This was utilized in exploratory experiments where pre-existing IgG against these antigens were measured. The results showed that IdeS treatment rapidly and effectively cleaved the antigen-specific IgG and that the reappearance of the antigen-specific IgG followed the reappearance of total IgG (Fig 6A and 6B) (individual results are shown in S1 Raw Data).

### Pharmacokinetics of IdeS

IdeS concentrations in serum were measured with selected reaction monitoring mass spectrometry, targeting four IdeS specific peptides derived from trypsin digested subject serum. The peptide concentration versus time data was analyzed by non-compartmental analysis. The pharmacokinetic parameters were calculated up to 24 hours post dosing, after which the serum concentrations were around or below the quantitative range of the method.

**Fig 7:**
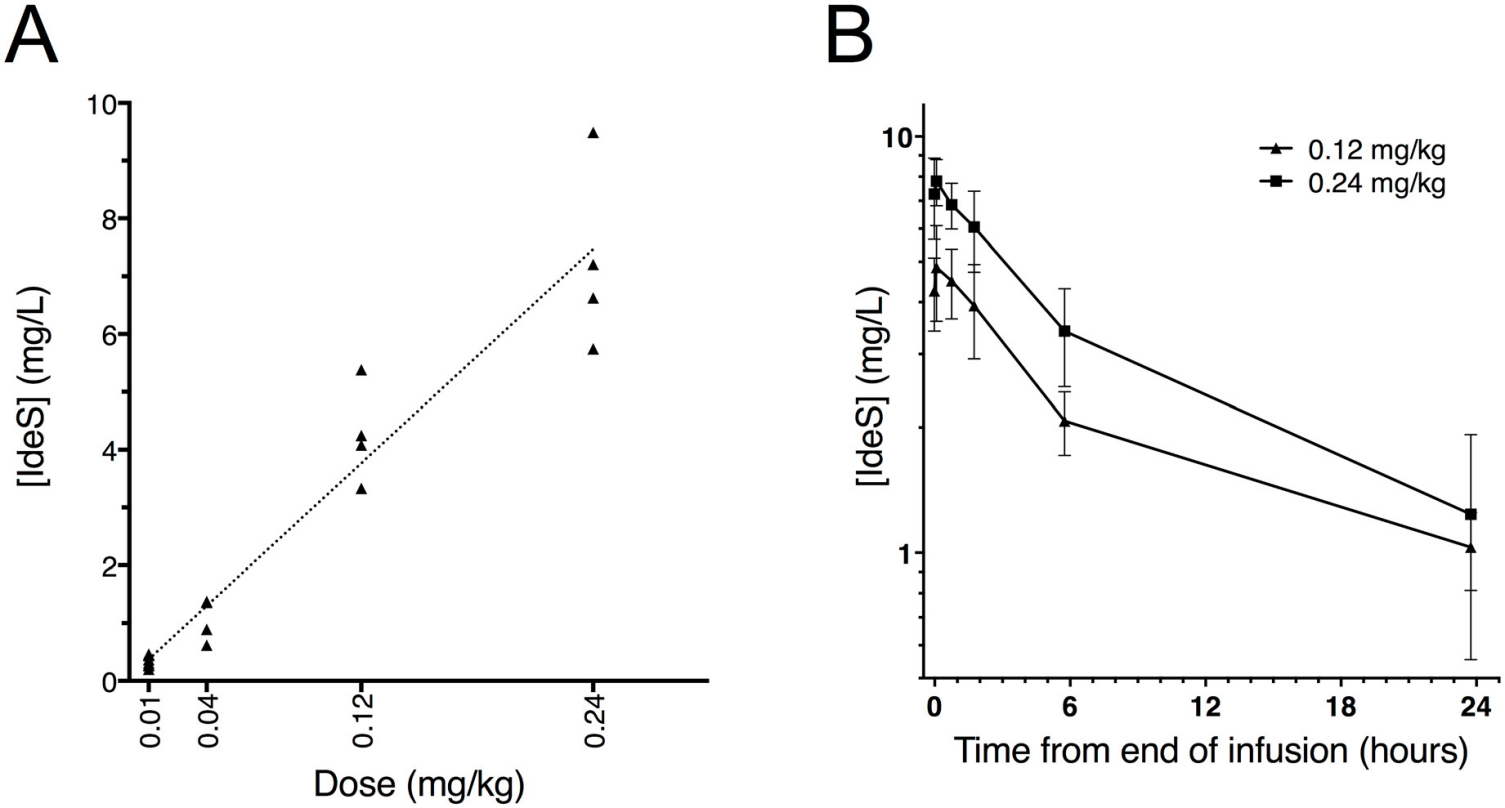
Pharmacokinetics of IdeS in serum. IdeS concentrations in serum samples from study subjects were determined by selected reaction monitoring mass spectrometry targeting four IdeS specific tryptic peptides. A) Comparison of serum IdeS concentration one minute before end of infusion versus dose levels of IdeS (0.01, 0.04, 0.12, and 0.24 mg/kg BW). Individual mean of two to four peptides. B) Comparison of serum concentration of mean values of two to four peptides versus time profiles up to 24 hours after infusion of 0.12 or 0.24 mg/kg BW IdeS (*n* = 8). Mean ± SEM.

Out of 29 subjects, nine received placebo and 20 IdeS in doses of 0.01, 0.04, 0.12 or 0.24 mg/kg BW. No IdeS derived peptides were detected in the pre-dose samples or in samples from the placebo subjects. However, IdeS was detected in samples from the IdeS-treated subjects, thereby confirming dosing. The IdeS concentration increased with dose and the increase in serum concentration was dose proportional (Fig 7A). As a consequence of only collecting PK samples at the one min prior to end of infusion time-point for the 0.01 and 0.04 mg/kg BW doses, further PK analysis was only possible for the 0.12 and 0.24 mg/kg BW dose groups.

In subjects dosed with 0.12 and 0.24 mg/kg BW, a total of 10 blood samples per subject were collected up to one week post dosing. The serum concentration of IdeS presents a multi-phase elimination curve where the main fraction was eliminated during the first 24 hours after dosing. During the first 6 hours after dosing, the mean t½ was 4.1 (±2.6) hours at 0.12 mg/kg BW and 4.9 (±2.8) hours at 0.24 mg/kg BW. The C_max_ was 5.0 (±2.5) mg/L at 0.12 mg/kg BW and 8.3 (±3.7) mg/L at 0.24 mg/kg BW (Fig 7B). Not all four peptides were measurable at every time point and the data shown in Fig 7B is a mean of at least two peptides for each point, however, in the majority of samples all four peptides were measurable (individual samples are shown in S1 Raw Data).

### IdeS treatment and phagocytic activity

**Fig 8:**
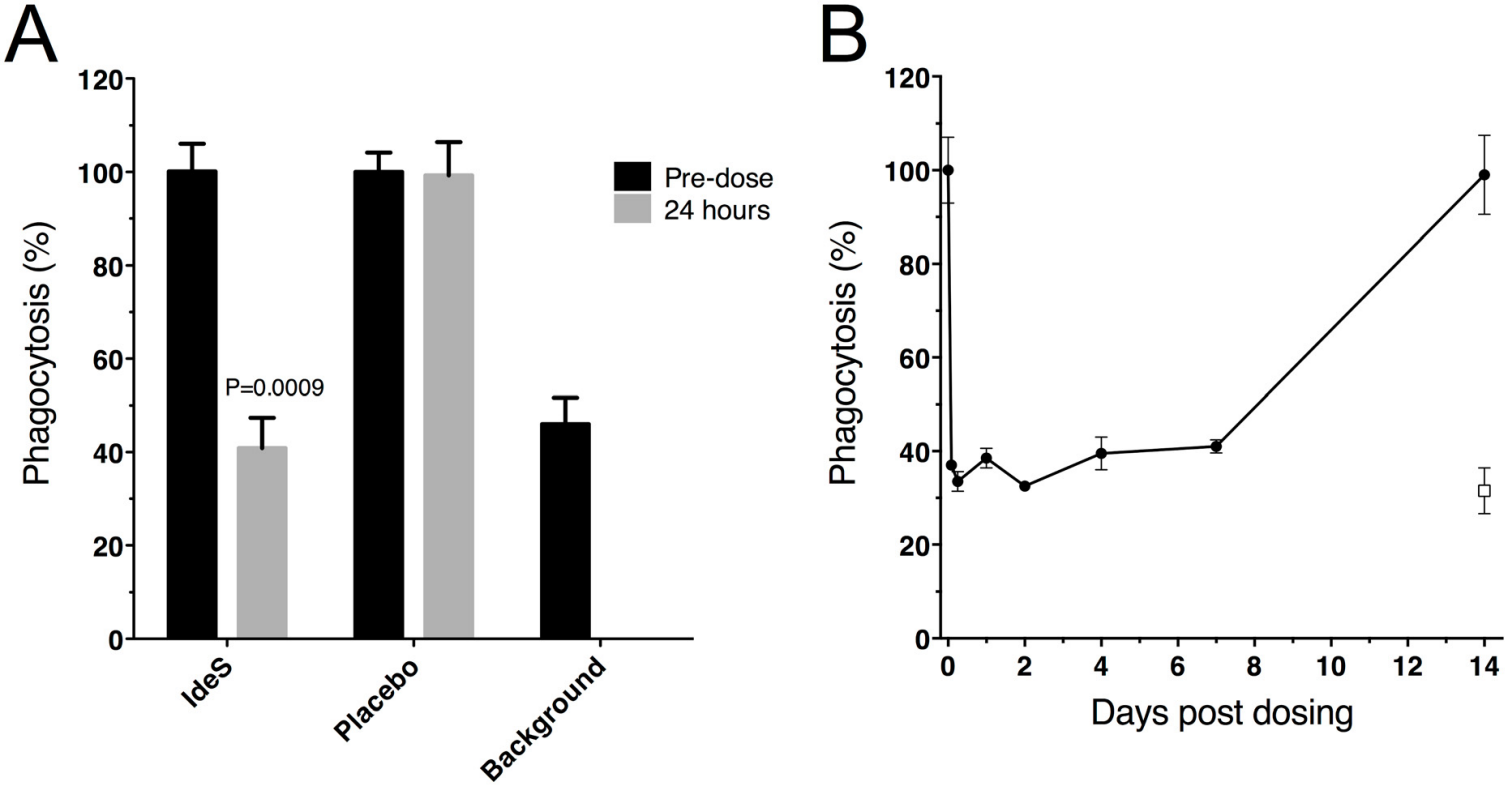
Serum from subjects dosed with IdeS showed impaired phagocytosis capacity. The opsonizing capacity of IgG in human serum was measured as percent of effector cells with at least one engulfed fluorescent serum-coated bead. Effector cells were gated and bead uptake was monitored as percent of cells shifted in the FL2 channel. Due to the extreme brightness of the fluorescent beads the signal could not me measured within the dynamic range of the FL1 channel and were instead monitored in FL2. A) Before and 24 hours after dosing of 0.24 mg/kg BW IdeS *vs*. placebo treated subjects. Pre-dose phagocytosis level for each individual was set to 100% and background is spontaneous uptake of beads in the absence of serum, *n* = 4 in the IdeS group and *n* = 2 in the placebo group. Mean of double wells/subject ± SD are shown. P-value was calculated using Mann-Whitney. B) Kinetics of the phagocytic potential in serum is shown for one representative subject in the 0.24 mg/kg BW group at different time-points (pre-dose, 2, 6, 24, 48 hours, 4, 7 and 14 days). Pre-dose phagocytosis level was set to 100% and background is spontaneous uptake of beads in the absence of serum (open box). Mean of double wells ± SD for this subject.

As an exploratory part of the study the functional activity of the remaining IgG was evaluated in a phagocytosis assay for the highest dose group. IgG from serum samples collected pre-dose and at different time-points after IdeS dosing were captured on fluorescent beads, washed, and mixed with effector cell. Phagocytosis was measured as percent of the effector cells with at least one phagocytized bead. Beads without serum represent the spontaneous uptake of non-opsonized beads by effector cells. All subjects dosed with 0.24 mg/kg BW reached background phagocytic levels 24 hours after dosing (Fig 8A) and had significantly decreased phagocytic capacity already at the two hour sampling point (individual results are shown in S1 Raw Data). The phagocytic capacity remained reduced for at least seven days (Fig 8B) after which it was again back at original levels when compared in a 100x diluted serum. This was a research assay with no formal lower limit of detection i.e. values should not be considered absolute. Several factors may impact the uptake of latex beads, the dilution factor of serum being one of them. Thus, a fixed dilution factor of 100 was determined to be a functional starting point for all pre-dose samples tested in this assay and this dilution was then used for all time-points. Another factor that may impact the uptake is blocking by the F(ab’)_2_ cleavage products. Interestingly, the day 7 sample on the gel in Fig 4C shows that the F(ab’)_2_ fragments are no longer present in circulation and there is still a reduced phagocytic potential (Fig 8B) indicating that the reduction is not caused by blocking F(ab’)_2_ fragments.

### IdeS and immunogenicity

**Fig 9:**
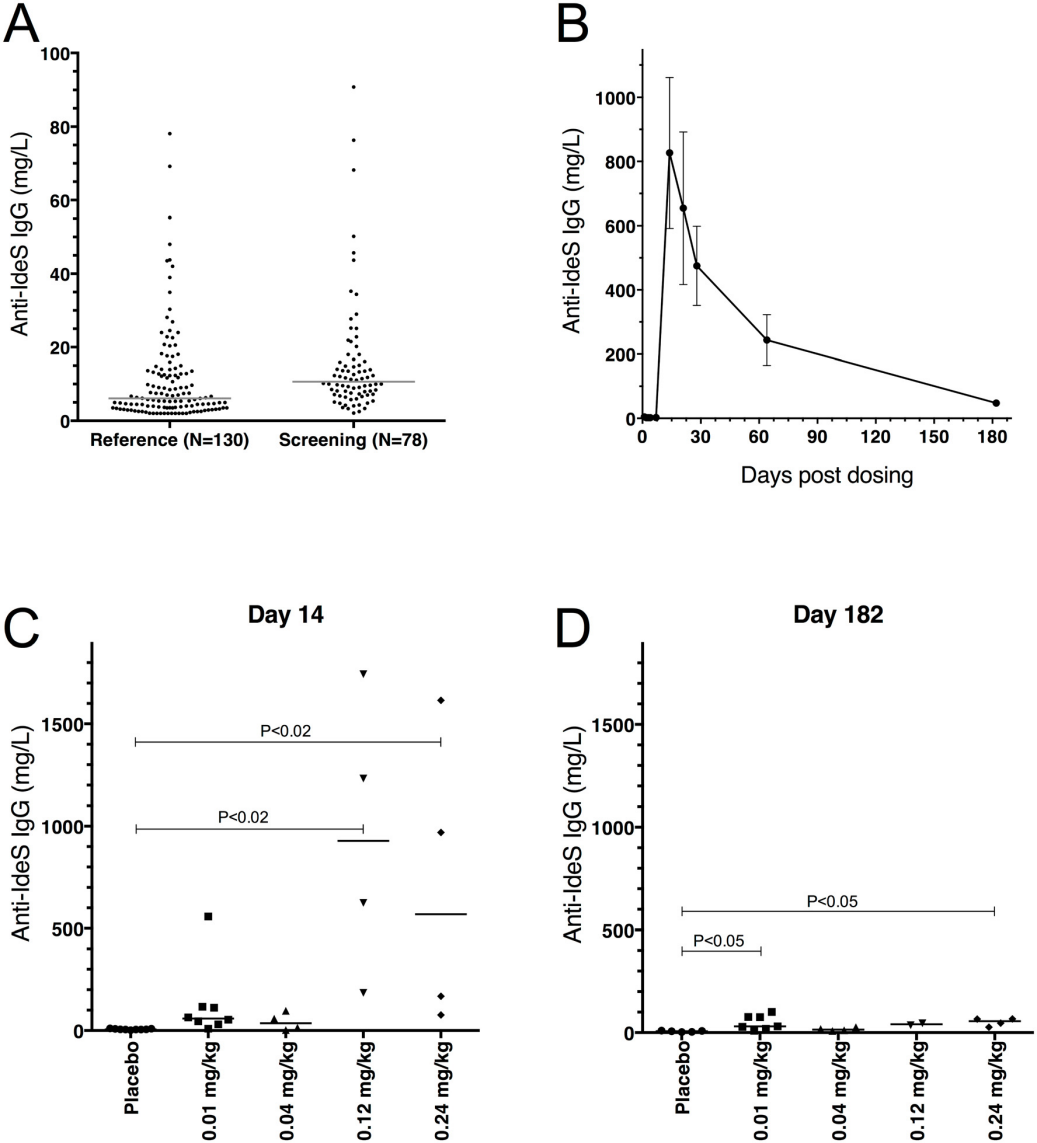
Anti-IdeS antibodies were followed before and throughout the study. Human serum samples were analyzed using the IdeS-ImmunoCAP (Thermo Fisher Scientific) on a Phadia 250 instrument. The cut off (LLOQ) for IgG was 2 mg/L. A) Samples from 130 human donors (reference) were compared to the 78 healthy human male subjects screened in this study (screening). The lines show median for the reference group (6.1 mg/L) and the screening group (10.6 mg/L). B) Kinetics of the anti-IdeS IgG levels shown as a mean for the 0.12 and 0.24 mg/kg BW groups (*n* = 8; mean ± SEM). No increase in anti-IdeS IgG is seen in any of the subjects prior to day 14. C) Anti-IdeS IgG levels shown for the separate groups at day 14 (*n*_Placebo_ = 9, *n*_0.01_ = 8, *n*_0.04_ = 4, *n*_0.12_ = 4 and *n*_0.24_ = 4), and D) at day 182 (*n*_Placebo_ = 5, *n*_0.01_ = 7, *n*_0.04_ = 4, *n*_0.12_ = 2 and *n*_0.24_ = 4). The subjects were asked to come back for an outside the study protocol sample on day 182. 17 out of 20 on active and 5 out of 9 on placebo volunteered. The lines show median level for each group. In C and D the groups were compared using Kruskal-Wallis, One-Way ANOVA, P = 0.0003 and P = 0.0082 respectively. The P-values shown in the graph represent comparisons of the mean rank of each dose-group with the placebo group using Dunn’s Multiple Comparison. The placebo group contains all subjects from all dose-groups treated with placebo.

A significant proportion of the population has pre-formed IgG antibodies against IdeS due to earlier *S*. *pyogenes* infections [17]. These anti-IdeS antibodies might increase the risk of hypersensitivity/infusion-like reactions against the drug IdeS. Therefore, a specific *in vitro* test system (IdeS-ImmunoCAP) for the quantitative measurement of IdeS specific antibodies was developed. Before inclusion subjects were screened by IdeS-ImmunoCAP and those with elevated IgG antibody titers (>15 mg/L) were excluded from the study. A reference group (*n* = 130) were screened with the IdeS-ImmunoCAP prior to study start. Ten out of 130 subjects had IdeS specific IgG below the cut-off (2.0 mg/L). The median level of anti-IdeS IgG was 6.1 mg/L (range: 2.0–78.0 mg/L; *n* = 130) with the 80% percentile at 15 mg/L. 78 healthy subjects were screened for this study and all had detectable IgG against IdeS. The median level of anti-IdeS IgG was 10.6 mg/L (range: 2.1–90.8 mg/L) and twenty-eight percent of the tested individuals had anti-IdeS IgG titers over 15.0 mg/L (Fig 9A).

The majority of the study subjects responded with an increase of anti-IdeS IgG. The response reached close to peak levels two weeks after dosing and then slowly decreased (Fig 9B). The median pre-dose level (all subjects) of anti-IdeS IgG was 5.3 mg/L (range: 2.0–10.6 mg/L), and on day 14 the median level of all subjects dosed with IdeS was 104.0 mg/L (range: 3.1–1744.0 mg/L). Two months after dosing the levels of anti-IdeS IgG had started to decrease and the median anti-IdeS IgG level of all subjects dosed with IdeS was 87.8 mg/L (range: 10.5–764.0 mg/L). Although the individual variation in the magnitude of the anti-IdeS IgG response was large, there was a significantly stronger response among the subjects receiving 0.12 or 0.24 mg/kg BW of IdeS compared to subjects receiving 0.01 or 0.04 mg/kg BW (Fig 9C). At day 182, the anti-IdeS IgG levels for 16 out of 17 IdeS receiving individuals were within the normal range of the previously analyzed subjects (i.e. 2.0–90.8 mg/L; *n* = 208) (Fig 9D) (individual results are shown in S1 Raw Data). Only one subject, receiving 0.01 mg/kg BW of IdeS, still had anti-IdeS IgG levels slightly above the normal range at day 182 (101.0 mg/L). Notably, the anti-IdeS IgG response was very similar in kinetics and magnitude to the response reported for other protein drugs of bacterial origin, such as streptokinase and staphylokinase [18–20].

## Discussion

Strict specificity for IgG and a rapid and efficient cleavage *in vitro*, early lead to the idea to use IdeS *in vivo* to incapacitate pathogenic IgG antibodies. Animal studies have demonstrated effective cleavage of IgG also *in vivo*, and the enzyme has a profound effect in animal models of IgG-driven autoimmune diseases. This study indicates that IdeS is safe and effective also in humans, suggesting that IdeS will prove to be a therapeutic option in several clinical conditions.

This first in class clinical phase I trial reveals that IdeS already minutes after administration converts plasma IgG into scIgG. ScIgG has compromised effector functions with reduced binding to Fcγ receptors and reduced Fc mediated cytotoxicity [21]. Despite the lack of pathogenic autoantibodies in healthy subjects, total plasma IgG and antigen-specific IgG could be measured as biomarkers, and IdeS showed impressive efficacy within the tested dose-range. Full or close to full effect on IgG, i.e. conversion into F(ab’)_2_ and Fc fragments, was seen in all subjects dosed with IdeS in doses 0.12 and 0.24 mg/kg BW, and the enzyme had a favorable safety profile. Six hours after administration only low concentrations of IgG could be detected in blood and IgG remained low for more than a week, until newly synthesized IgG appeared in plasma. This demonstrates that the entire extracellular IgG pool and not only the plasma pool, is cleaved by IdeS. The remarkable efficacy of the protease outcompetes the effect of a single plasma volume exchange, which typically leaves 35% remaining IgG and 24 hours thereafter the levels have raised to 60%, mainly due to lymphatic drainage into the vascular space [22]. Since *S*. *pyogenes* is a common human pathogen all subjects had pre-formed anti-IdeS IgG antibodies and reacted as expected with an IgG response peaking two to three weeks after the IdeS infusion. The amplitude of the anti-drug response varied substantially between individuals, although a dose-response pattern was noted. Six to twelve months after dosing all subjects were back to normal anti-IdeS antibody levels (i.e. <2–91 mg/L). Considering safety and potentially neutralizing antibodies it is anticipated that IdeS treatment could be repeated after six to twelve months in accordance with the experience from e.g. streptokinase treatment [23]. The IdeS specific ImmunoCAP test, developed in parallel with our clinical studies, may prove to be a valuable tool for clinicians considering repeated dosing.

In addition to total plasma IgG we investigated the effect of IdeS on antigen-specific IgG. Pentavac, a vaccine against diphtheria, tetanus, pertussis, polio and *Haemophilus influenzae* type b, is utilized as a vaccine within the Swedish childhood vaccination program and, consequently, all tested subjects had pre-formed IgG against the vaccine components. The results showed no significant difference in the cleavage and recovery of the IgG antibodies against these antigens compared to total IgG; all subjects had fully recovered their antigen-specific IgG at the time when the IgG pool was back to pre-dose levels.

The functional relevance of cleaving IgG with IdeS was evaluated in a phagocytosis assay, where interaction with the Fcγ receptor is expected to play a major role. Only a few hours post administration of 0.24 mg/kg BW IdeS, the phagocytic effect of remaining IgG/IgG fragments was significantly reduced in all tested subjects, an effect remaining seven days later. Importantly, IdeS had the capacity to inactivate Fc-mediated effector function *in vivo* in humans.

IdeS is currently considered for clinical studies within several acute antibody mediated conditions such as antibody mediated graft rejection, Guillain-Barrés syndrome and Goodpastures syndrome. A phase II clinical study in sensitized dialysis patients, is currently running in Sweden. Approximately one third of patients waiting for kidney transplantation are sensitized to human major histocompatibility complex (MHC) antigens [24]. Antibodies are developed as a consequence of previous transplantations, blood transfusions, or pregnancies. Sensitization to donor MHC hampers the identification of a suitable donor. Approximately 15% of the listed patients are classified as highly sensitized. High titers of donor specific antibodies is a direct contraindication to transplantation because of the high risk of acute antibody-mediated rejection. Plasma exchange or immune adsorption often in combination with intravenous gamma globulin or rituximab is an accepted strategy to lower the levels of donor specific antibodies to a level where transplantation can be considered [25–28]. The disadvantages with plasma exchange, immune adsorption and intravenous gamma globulin treatments are inefficiency and the requiring of rigorous planning since they involve repeated treatments over an extended time period. A kidney from a deceased donor has to be transplanted within hours since prolonged cold ischemia time is an important risk factor for delayed graft function and allograft loss [29]. IdeS treatment has the capacity to quickly and effectively remove IgG in the sensitized patient, thereby allowing transplantation and avoiding acute antibody-mediated rejection. IdeS safety and efficacy in removing anti-MHC antibodies in sensitized dialysis patients are currently investigated in an ongoing phase II study.

In summary, a single dose of IdeS rapidly and efficiently inactivates IgG in humans and the effect remains for several weeks. IdeS alone and/or in combination with B-cell attenuating drugs (e.g. bortezomib or rituximab) is a very attractive therapeutic approach for many IgG driven conditions. The immunogenic nature of a bacterial protein like IdeS presumably prevents chronic treatment although repeated dosing once or twice a year might be possible. However, by utilizing the high efficacy of IdeS in combination with other drugs or technologies such as immune adsorption or plasma exchange, it should be possible to maintain low plasma levels of pathogenic antibodies for an extended period of time.

The demonstration that a virulence factor of one of the most significant bacterial pathogens in the human population can be safely administered to humans is in itself noteworthy. In addition, the remarkable speed and efficacy of IgG neutralization, as well as the duration of the effect, makes IdeS treatment superior to other IgG removing options like plasma exchange and immune adsorption. In *S*. *pyogenes*, IdeS has evolved to disarm host IgG and thereby protect the bacteria against the human immune system. The present investigation provides the fascinating and realistic possibility that this bacterial enzyme will be used in the future to treat patients with severe IgG-driven diseases.

## Supporting Information

S1 Protocol The study protocol. A phase I single centre study to evaluate the safety, tolerability and pharmacokinetics of intravenous IdeS after administration of single ascending doses in healthy male subjects.
(PDF)

S1 Checklist The CONSORT checklist. (DOC)

S1 Raw Data Raw data for Fig 2 and Figs 5–9. (XLSX)

S1 Fig Phagocytosis assay gating. The left plot shows the forward and side scatter of the fluorescent beads. The middle plot shows the scatter for the THP-1 cells and the right plot shows the scatter for cells and beads when mixed together. Cells are clearly separated from beads and the gated area (R1) is shown in the plots.
(TIF)

S2 Fig Phagocytosis assay kinetics. Cells were gated in R1 (see S1 Fig) and further monitored for bead uptake in FL2. The graphs show the phagocytic potential of IgG and IgG-fragments present in serum collected at different time-points post dosing of 0.24 mg/kg BW of IdeS. Above each graph is the time-point post dosing shown, with pre-dose in the upper left corner. The last plot shows background which is bead uptake in the absence of serum.
(TIF)
