# Supplementary material for: Complete Removal of Extracellular IgG Antibodies in a Randomized Dose-Escalation Phase I Study with the Bacterial Enzyme IdeS – A Novel Therapeutic Opportunity
Source: PLoS One. 2015 Jul 15;10(7):e0132011. doi: 10.1371/journal.pone.0132011 (PMC4503742; doi:10.1371/journal.pone.0132011)
Supplement: S1 Protocol — A phase I single centre study to evaluate the safety, tolerability and pharmacokinetics of intravenous IdeS after administration of single ascending doses in healthy male subjects. (PDF) [file pone.0132011.s001.pdf]

A PHASE I SINGLE CENTRE STUDY TO EVALUATE THE SAFETY, TOLERABILITY AND PHARMACOKINETICS OF INTRAVENOUS IDES AFTER ADMINISTRATION OF SINGLE ASCENDING DOSES IN HEALTHY MALE SUBJECTS

Sponsor: Hansa Medical AB, Lund, Sweden

Eudract No: 2012-000 969-21

Strictly confidential. This document contains confidential information belonging to Hansa Medical AB

SIGNATURE PAGE

Lena Winstedt, PhD
Clinical Research Manager
Hansa Medical AB
Scheelevägen 22, Lund
Sweden

20 MAR 2013

Signature Date

Christian Kjellman, PhD
Chief Scientific Officer
Hansa Medical AB
Scheelevägen 22, Lund
Sweden

20 Mars 2013

Signature Date

Ulf Malmqvist, MD
Principle investigator
Clinical Trials Unit, R&D Centre
Skåne University Hospital, Lund
Sweden

Signature Date

Fredrik Hansson
Statistician
Commitum/Norma
Traktorvägen 11, Lund
Sweden

Signature Date

Gunnar Tufveson, MD, PhD
Professor of Transplant Surgery
Study team physician
Dept. of Transplant Surgery
Uppsala University Hospital
Sweden

Signature Date

Strictly confidential. This document contains confidential information belonging to Hansa Medical AB

SIGNATURE PAGE

Lena Winstedt, PhD
Clinical Research Manager
Hansa Medical AB
Scheelevägen 22, Lund
Sweden

Signature Date

Christian Kjellman, PhD
Chief Scientific Officer
Hansa Medical AB
Scheelevägen 22, Lund
Sweden

Signature Date

Ulf Malmqvist, MD
Principle investigator
Clinical Trials Unit, R&D Centre
Skåne University Hospital, Lund
Sweden

29/03/2013

Signature Date

Fredrik Hansson
Statistician
Commitum/Norma
Traktorvägen 11, Lund
Sweden

Signature Date

Gunnar Tufveson, MD, PhD
Professor of Transplant Surgery
Study team physician
Dept. of Transplant Surgery
Uppsala University Hospital
Sweden

Signature Date

Strictly confidential. This document contains confidential information belonging to Hansa Medical AB

SIGNATURE PAGE

Lena Winstedt, PhD
Clinical Research Manager
Hansa Medical AB
Scheelevägen 22, Lund
Sweden

Signature Date

Christian Kjellman, PhD
Chief Scientific Officer
Hansa Medical AB
Scheelevägen 22, Lund
Sweden

Signature Date

Ulf Malmqvist, MD
Principle investigator
Clinical Trials Unit, R&D Centre
Skåne University Hospital, Lund
Sweden

Signature Date

Fredrik Hansson
Statistician
Commitum/Norma
Traktorvägen 11, Lund
Sweden

Signature Date

Gunnar Tufveson, MD, PhD
Professor of Transplant Surgery
Study team physician
Dept. of Transplant Surgery
Uppsala University Hospital
Sweden

2013-03-25

Signature Date

SIGNATURE PAGE

Lena Winstedt, PhD
Clinical Research Manager
Hansa Medical AB
Scheelevägen 22, Lund
Sweden

Signature Date

Christian Kjellman, PhD
Chief Scientific Officer
Hansa Medical AB
Scheelevägen 22, Lund
Sweden

Signature Date

Ulf Malmqvist, MD
Principle investigator
Clinical Trials Unit, R&D Centre
Skåne University Hospital, Lund
Sweden

Signature Date

Fredrik Hansson
Statistician
Commitum/Norma
Traktorvägen 11, Lund
Sweden

22 MAR 2013

Signature Date

Gunnar Tufveson, MD, PhD
Professor of Transplant Surgery
Study team physician
Dept. of Transplant Surgery
Uppsala University Hospital
Sweden

Signature Date

TABLE OF CONTENT

TABLE OF CONTENT 3

1 LIST OF ABBREVIATIONS AND DEFINITION OF TERMS 6

1 IMPORTANT MEDICAL PROCEDURES TO BE FOLLOWED BY THE INVESTIGATOR 8
1.1 MEDICAL EMERGENCIES CONTACTS 8
1.2 OVERDOSE 8

2 INTRODUCTION 9
2.1 RATIONALE FOR CONDUCTING THIS STUDY 9
2.1.1 Disease and patient population 9
2.1.2 Current treatments 10
2.1.3 IdeS 11
2.1.4 Rationale for overall study design and dose groups 12
2.2 BENEFIT/RISK AND ETHICAL ASSESSMENT 13

3 STUDY OBJECTIVES 14
3.1 PRIMARY OBJECTIVE 14
3.2 SECONDARY OBJECTIVES 14

4 STUDY PLAN AND PROCEDURES 14
4.1 OVERALL STUDY DESIGN AND FLOW CHART 14
4.1.1 Stopping criteria for dose escalation 19
4.1.2 Definition of dose limiting toxicity (DLT) 20
4.1.3 Data Monitoring Committee (DMC) 20

5 SUBJECT SELECTION CRITERIA 21
5.1 INCLUSION CRITERIA 21
5.2 EXCLUSION CRITERIA 21
5.3 WITHDRAWAL OF SUBJECTS 23
5.3.1 Criteria for discontinuation from the study 23
5.3.2 Procedures for discontinuation of a subject from the study 23

6 STUDY CONDUCT 23
6.1 RESTRICTIONS DURING THE STUDY 23
6.2 SUBJECT ENROLMENT 24
6.3 STUDY TREATMENT 24
6.3.1 IdeS 24
6.3.2 Doses and treatment regimens 25
6.4 CONCOMITANT AND POST-STUDY TREATMENT(S) 26
6.4.1 Prophylactic antibiotics 26
6.4.2 Other concomitant medication 26
6.5 TREATMENT COMPLIANCE 26
6.5.1 Accountability 26

7 STUDY ASSESSMENTS 27
7.1 RECORDING OF DATA 27
7.2 SCREENING AND DEMOGRAPHY PROCEDURES 27

7.2.1 Follow-up procedures *28*
7.3 SAFETY 28
7.3.1 Definition of adverse events *29*
7.3.2 Definitions of serious adverse event *29*
7.3.3 Recording of adverse events *29*
7.3.4 Variables *29*
7.3.5 Adverse Events based on signs and symptoms *30*
7.3.6 Adverse Events based on examinations and tests *30*
7.3.7 Follow-up of unresolved adverse events *30*
7.3.8 Reporting of serious adverse events *30*
7.3.9 Reporting of suspected unexpected serious adverse events (SUSARs) *31*
7.3.10 Laboratory safety assessment *31*
7.3.11 Physical examination *33*
7.3.12 Height and weight *33*
7.3.13 ECG *33*
7.3.14 Vital signs *33*
7.4 PHARMACOKINETICS AND PHARMACODYNAMICS 33
7.4.1 Collection of biological samples *33*
7.4.2 Determination of drug concentration in biological samples *34*
7.4.3 Determination of IgG levels in serum *34*
7.5 ANTI-DRUG ANTIBODY ANALYSIS 34
7.5.1 Collection of biological samples *34*
7.5.2 Determination of ADA levels in serum *34*
7.6 EXPLORATORY BIOMARKER ANALYSIS 34
7.6.1 Determination of anti-tetanus and anti-CMV levels in serum *34*
7.6.2 Determination of Fc and Fab in serum *34*
7.6.3 Detection of B cell receptor cleavage in vivo *34*

8 BIOLOGICAL SAMPLING PROCEDURES 35
8.1 VOLUME OF BLOOD 35
8.2 HANDLING, STORAGE AND DESTRUCTION OF BIOLOGICAL SAMPLES 35
8.2.1 Pharmacokinetic, pharmacodynamic and anti-drug antibody samples *35*
8.3 CHAIN OF CUSTODY OF BIOLOGICAL SAMPLES 35
8.4 WITHDRAWAL OF INFORMED CONSENT FOR DONATED BIOLOGICAL SAMPLES 35

9 ETHICAL AND REGULATORY REQUIREMENTS 36
9.1 ETHICAL CONDUCT OF THE STUDY 36
9.2 SUBJECT DATA PROTECTION 36
9.3 ETHICS AND REGULATORY REVIEW 36
9.4 INFORMED CONSENT 37
9.5 CHANGES TO THE PROTOCOL AND INFORMED CONSENT FORM 37
9.6 AUDITS AND INSPECTIONS 38

10 STUDY MANAGEMENT 38
10.1 PRE-STUDY ACTIVITIES 38
10.2 TRAINING OF STUDY SITE PERSONNEL 38
10.3 MONITORING OF THE STUDY 38
10.3.1 Source data *39*
10.4 STUDY AGREEMENTS 39

10.5 STUDY TIMETABLE AND END OF STUDY 39

11 DATA MANAGEMENT 39

12 EVALUATION AND CALCULATION OF VARIABLES 40

12.1 CALCULATION OR DERIVATION OF PHARMACOKINETIC VARIABLES 40

12.2 CALCULATION OR DERIVATION OF PHARMACODYNAMIC VARIABLES 41

12.2.1 Calculation or derivation of the relationship between pharmacokinetic and pharmacodynamic variables 41

13 STATISTICAL METHODS AND SAMPLE SIZE 41

13.1 DESCRIPTION OF ANALYSIS SETS 41

13.1.1 General principles 41

13.2 METHODS OF STATISTICAL ANALYSES 41

13.2.1 General principles 41

13.2.2 Subject characteristics 41

13.2.3 Safety and tolerability 41

13.2.4 Pharmacokinetics 42

13.2.5 Pharmacodynamics 42

13.3 DETERMINATION OF SAMPLE SIZE 42

14 REFERENCES 42

1 LIST OF ABBREVIATIONS AND DEFINITION OF TERMS

The following abbreviations and special terms are used in this study protocol.

Abbreviation or special term	Explanation
ADA	Anti drug antibody
AE	Adverse event
ALT	Alanine aminotransferase
AMR	Antibody mediated rejection
AST	Aspartate aminotransferase
AUC	Area under serum concentration-time curve
BMI	Body mass index
BP	Blood pressure
BW	Body weight
CDC	Complement depending cytotoxicity
CKD	Chronic kidney disease
CL	Clearance of drug from plasma/serum
CL/F	Total apparent clearance of drug from plasma/serum
$C_{max} = C_{0hr}$	Maximum serum concentration
CMV	Cytomegalovirus
CRF	Case report form (electronic/paper)
CRM	Clinical research manager
CRO	Contract research organisation
CTCAE	Common toxicity criteria for adverse events
CV	Coefficient of variation
DCF	Data clarification form
DLT	Dose limiting toxicity
DMC	Data monitoring committee
DSA	Donor specific antibodies
EC	Ethics committee synonymous to Independent Ethics committee (IEC)
ECG	Electrocardiogram
GCP	Good clinical practice
Hb	Hemoglobin
HIV	Human immunodeficiency virus

Abbreviation or special term	Explanation
HLA	Human leukocyte antigen
IA	Immuno adsorption
ICH	International conference on harmonisation
IdeS	Immunoglobulin G-degrading enzyme of *Streptococcus pyogenes*
IVIG	Intravenous immunoglobulin
MABEL	Minimal anticipated biological effect level
MED	Minimal effective dose
MedDRA	Medical dictionary for regulatory activities
MTD	Maximum tolerated dose
MRT	Maximum residence time extrapolated to infinity
NIH	National institute of health
Nmiss	Number of missed observations
NOAEL	No observed adverse effect level
Nobs	Number of observations
OBD	Optimal biological dose
OPTN	Organ procurement and transplantation database
PD	Pharmacodynamics
PI	Principal investigator
PK	Pharmacokinetics
PRA	Panel reactive antibody
PSA	Prostate specific antibody
QC	Quality control
SAD	Single ascending dose
SAE	Serious adverse event
SAP	Statistical analysis plan
SD	Standard deviation
SMD	Study maximal dose
SOC	System organ class
SOP	Standard operating procedure
Ss	Steady state
SUSAR	Suspected unexpected serious adverse events

Strictly confidential. This document contains confidential information belonging to Hansa Medical AB

Abbreviation or special term	Explanation
Tau	Dosing interval
$t_{½}$	Terminal half-life
t_{max}	Time to maximum plasma concentration
ULN	Upper limit of normal
V_z	Apparent volume of distribution
V_{ss}	Volume of distribution based at steady state

1 IMPORTANT MEDICAL PROCEDURES TO BE FOLLOWED BY THE INVESTIGATOR

1.1 Medical emergencies contacts

The principal investigator is responsible for ensuring that procedures and expertise are available to handle medical emergencies during the study. **A medical emergency usually constitutes an SAE and is to be reported as such.**

In the case of a medical emergency the investigator may contact the Clinical Research Manager at Hansa Medical.

Name	Role in the study	Address & telephone number
Lena Winstedt	Clinical Research Manager	+46 768 58 15 06<br>lena.winstedt@hansamedical.com
Ulf Malmqvist	Principal investigator	+46 46 17 33 33<br>ulf.malmqvist@skane.se
Gunnar Tufveson	Study team physician	+46 708 27 67 68<br>gunnar.tufveson@akademiska.se
Per Åkesson	Study team physician<br>(Specialist in infectious diseases)	+46 708 60 03 25<br>per.akesson@skane.se

1.2 Overdose

An overdose is a dose in excess of the dose specified for each cohort of the protocol. There are no data on overdosing since this is the first study in humans. There is no known antidote but depletion of IgG can be restored with intravenous gammaglobulin. In the event of an overdose the subject should be monitored closely and treated symptomatically. This should be recorded as follows:

- An overdose with associated AEs is recorded as the AE diagnosis/symptoms on the relevant AE modules in the CRF
- An overdose without associated symptoms is only reported in the subject file

2 INTRODUCTION

2.1 Rationale for conducting this study

2.1.1 Disease and patient population

Graft rejections after solid organ transplantation can be divided into two different types, cellular rejection (lymphocyte mediated) and humoral or antibody mediated rejection (AMR) (Chinen and Buckley, 2010; Colvin, 2007; Lucas *et al.*, 2011). AMR occurs in up to 20-30% of all acute rejections following kidney transplantation and can be the single cause of a rejection event or interact with cellular rejection (Lucas *et al.*, 2011). AMR is divided into three different groups depending on when in time the rejection reaction occurs. Hyperacute rejection occurs within minutes to hours after transplantation. Acute AMR occurs within a few days following transplantation and can involve T-cell reactivity as well as antibodies. Chronic AMR occurs after months or years and involves both T-cells and antibodies (Archdeacon *et al.*, 2011; Colvin and Smith, 2005).

Chronic kidney disease (CKD) stage 5 is the most severe grade of CKD and means a complete loss of renal function. Renal transplantation is the preferred treatment choice for patients with CKD stage 5 and transplantation increases survival and patient quality of life and results in substantial savings in health care costs compared to dialysis (Montgomery *et al.*, 2005). Data from the Eurotransplant and the Scanditransplant databases reveal that in 2009, 9 983 solid organ transplantations were performed in the member countries. Out of these 58% or 5776 patients were kidney transplantations.

Transplantation in the presence of donor specific antibodies (DSA) risk resulting in a hyperacute antibody-mediated rejection with acute allograft loss. However, since the introduction of the cross-match test, where the recipients serum is tested for cytotoxicity against lymphocytes from the potential donor, no transplantations are performed against a positive cross-match and hyper-acute rejections are extremely rare (Patel and Terasaki, 1969). About 20-40% of patients on the renal transplantation waiting list in US and EU have pre-formed alloantibodies usually to human leukocyte antigens (HLA) class I and/or class II, and sensitization constitutes a major problem in renal transplantation (Moll and Pascual, 2005; Montgomery and Zachary, 2004; Susal and Morath, 2011; Terasaki and Ozawa, 2004). The anti-HLA antibodies originate from allogenically sensitized B cells and are usually present in patients that have previously been sensitized by blood transfusion, previous transplantation or pregnancy (Jordan *et al.*, 2003). Sensitized patients have a prolonged waiting time, a reduced transplant rate and an increased risk of death and several studies particularly in renal transplantation have concluded that sensitization to donor HLA represents an unequivocal contra-indication to transplantation (Colvin, 2007; Gebel *et al.*, 2003; Gloor *et al.*, 2008; Montgomery *et al.*, 2011). The consequence of a positive cross-match test being an unambiguous barrier to transplantation is an accumulation of sensitized patients on the wait-list and the median waiting time in the US for renal transplant recipients listed in 2001–2002 was 1329 days for non-sensitized patients (PRA 0–9%), 1920 days for slightly to moderately sensitized patients (PRA 10–79%), and 3649 days for highly sensitized patients (PRA ≥80%)(OPTN-database, 2011)(http://optn.transplant.hrsa.gov/).

Sensitized patients have an increased risk of AMR associated with a negative short- and long-term graft outcome (Kissmeyer-Nielsen *et al.*, 1966; Lefaucheur *et al.*, 2007; Lefaucheur *et al.*, 2008). However, in a recent study it was shown that candidates for kidney transplantation being donor specific HLA-antibody positive benefit from donor specific antibody removal by plasmapheresis and low dose IVIG followed by living donor transplantation compared to staying on dialysis waiting for an HLA-compatible kidney. The patient survival at 5 years was 81% after removal of donor specific antibodies followed by transplantation compared to 51% when staying on dialysis. At 8 years the corresponding numbers were 81% compared to 31% patient survival (Montgomery *et al.*, 2011). Thus, a treatment that reduces antibody levels to enable transplantation resulted in improvements in survival and life quality as well as significant reductions of health care costs for this group of patients.

2.1.2 Current treatments

Although there is currently no consensus on treatment regimens to reduce pre-formed donor specific antibodies in patients, a few experimental methods have been tested in clinical settings.

Immunoadsorption (IA) has, in small experimental studies, showed its capacity in lowering anti-HLA antibodies and rendering a positive pre-transplant cross-match negative (Higgins *et al.*, 1996). In a small series of 13 deceased donor kidney recipients with a positive cross-match prior to transplantation IA treatment immediately before transplantation resulted in a negative cross-match. At latest follow-up (median 26 months, range 9-42), 12 of 13 patients were alive and seven of 13 grafts were functional with a median plasma creatinine concentration of 185 micromol/L (range 106-296). There was no graft loss as a result of classic hyperacute rejection.

In addition, over the past several years, two regimens have evolved to prevent antibody-mediated rejection. These are the high-dose intravenous immune globulin (IVIG) protocol and the plasmapheresis plus low-dose IVIG protocol (Gloor *et al.*, 2003; Jordan *et al.*, 2004; Jordan *et al.*, 2003; Jordan *et al.*, 2006). Protocols for plasmapheresis and low-dose IVIG were first used in 1998 to reduce anti-HLA donor-specific antibodies to allow transplantation across a positive cross-match (Gloor *et al.*, 2003; Montgomery and Zachary, 2004). Plasmapheresis has a potential benefit by the way of antibody clearance (Akalin *et al.*, 2008) and the protocols produce reduction in anti-HLA titers that allows for transplantation after four to five plasmapheresis treatments depending on the pre-treatment level of anti-HLA antibodies. The addition of low-dose IVIG is considered to add an immunomodulatory effector mechanism that benefits in keeping antibody titers low.

Only one randomized, placebo-controlled trial in moderately to highly sensitized patients (PRA $\geq$50%) has been conducted by the National Institute of Health (NIH) (Jordan *et al.*, 2004). The protocol used consisted of four consecutive monthly administrations of IVIG at a dose of 2 g/kg. A total of 101 adult patients with chronic kidney disease who were highly sensitized to HLA antigens were enrolled into the trial. In this study IVIG significantly reduced the anti-HLA antibody level (p=0.04) and improved the rate of transplantations as compared to placebo (35% *vs*. 17%, p=0.02).

Plasmapheresis, immunoadsorption and IVIG repeat dosing all have the disadvantage of requiring rigorous planning since it involves repeated treatments over a long period of time. When an organ from a deceased donor becomes available it has to be transplanted within hours since prolonged cold ischemia time is one of the most important risk factor for delayed graft function and allograft loss in renal transplantation (Ojo *et al.*, 1997). Therefore, neither of these treatments are options for patients receiving organs from a deceased donor.

Other treatment schedules used to reduce DSA levels include low dose IVIG + rituximab (Reinsmoen *et al.*, 2008), IVIG+Thymoglobulin (Akalin *et al.*, 2008; Bächler *et al.*, 2010; Mai *et al.*, 2009).

In a recent study from Heidelberg (Morath *et al.*, 2012), 10 CDC and ELISA cross-match positive patients received a living donor kidney transplant after desensitization with multiple pre-operative immunoadsorption (IA) treatments making CDC and ELISA cross-matches negative. Before desensitization, median levels of mean fluorescent intensity (MFI) measured by Luminex was 6203, which decreased to 891 MFI immediately before transplantation. After transplantation, IA treatment was continued until good allograft function was achieved and DSA was on acceptable levels (below 1000 MFI). Patients also received anti-CD20 (rituximab) and basiliximab (or thymoglobuline) induction, tacrolimus, EC-MPS and steroids. Post-operative DSA and kidney allograft biopsies were monitored and the patients had repeated IA-treatments if DSA returned. Two patients had acute cell-mediated rejection and 3 had AMR, the latter were treated with daily apheresis and repeated rituximab. Results were excellent with 100% 2-year graft survival and median serum creatinine of 1.6 mg/dl. An important observation was that in 6 patients the DSA remained below 1000 MFI (as measured by Luminex) during the entire follow up.

Taken together, experience with desensitization of patients with DSA have shown that results are similar to those in non-sensitized patients and that the patients have a substantial chance of long-term graft survival (although numbers are small and studies are not randomized). However, the methods currently used are time-consuming and incomplete and therefore not suitable for the deceased donor setting. Therefore, better alternatives are needed for these patients who can otherwise not be transplanted.

2.1.3 IdeS

Immunoglobulin G-degrading enzyme of *Streptococcus pyogenes* (IdeS) is a cysteine protease and an IgG endopeptidase originating from *Streptococcus pyogenes* MGAS5005 (Taxonomy ID: 293653). The active substance consists of a recombinant protein where amino acid 30-339 of the IdeS molecule, expressed in *Escherichia coli* BL21 (DE) from the pET9a expression vector, has been purified using a series of chromatography steps. IdeS neutralizes IgG with high efficacy *in vitro* and *in vivo* in animals and cleaves all subclasses of human IgGs just downstream of the hinge region, generating one $F(ab')_2$ and one homodimeric Fc fragment (HMed Doc. No. 2012-003; Johansson *et al.*, 2008; Nandakumar *et al.*, 2007; Wenig *et al.*, 2004; Vincents *et al.*, 2004; von Pawel-Rammingen *et al.*, 2002) The proteolytic activity of IdeS is dependent on both physical interaction with the IgG Fc-domain C_H2 and C_H3 and on the recognition of a target sequence located just C-terminally of the IgG hinge region

(Agniswamy *et al.*, 2004; Wenig *et al.*, 2004; Vincents *et al.*, 2004) where IdeS cleaves after Gly236 residue.

IdeS efficiently cleaves IgG from human and rabbit, but has no or poor activity on IgG from all other tested species such as dog, mouse, rat, pig, marmoset, Cynomolgus monkey and Rhesus macaque (HMed Doc. No. 2012-003; Agniswamy *et al.*, 2004; Nandakumar *et al.*, 2007). Additional work performed by Hansa Medical AB (HMed Doc. No. 2012-003) clearly demonstrate that IdeS efficiently cleaves purified IgG as well as IgG in serum from human and rabbit and that the enzyme is particularly active in rabbit where very low concentrations of IdeS can cleave > 99% of the IgG.

IdeS treatment can rapidly and substantially reduce the level of total IgG *ex vivo* in human serum samples. Furthermore, this activity is directly reflected as a reduction of anti-HLA IgG in serum from sensitized CKD grade 5 patients. Luminex analyses clearly demonstrate that IdeS treatment has the capacity to reduce the reactivity to individual MHC-antigens below the critical MFI for transplantation, i.e. below 1000 and to turn a positive cross-match into a negative. This is a strong indication that IdeS treatment just prior to transplantation has the potential to desensitize a highly sensitized patient and thereby allowing transplantation and avoiding a hyperacute antibody mediated rejection.

2.1.4 Rationale for overall study design and dose groups

This single ascending dose (SAD), double blind, randomised study is the first study in man with IdeS and it is designed to evaluate the safety, tolerability, pharmacokinetics (PK) and pharmacodynamics (PD) of IdeS in healthy male subjects after a single intravenous dose. It will include up to 36 healthy male subjects in up to six dose groups. Each group will comprise six subjects (4 on IdeS and 2 on placebo). The results from this study will form the basis for the design of a study in the intended patient population. This study in healthy subjects will also have the advantage that a dose response relationship can be measured *in vivo* in humans since IgG degradation can be closely monitored throughout the study period. Subjects will be given single intravenous predefined doses of IdeS or placebo. In the first dose group the dose is 0.010 mg/kg body weight (BW).

The starting dose for this first clinical study in humans has been calculated based on the minimum anticipated biological effect level (MABEL) in humans and the no observed adverse effects level (NOAEL) in the toxicology species. It is set to 0.010 mg/kg BW, which is ten times below the human MABEL and 200 times below the NOAEL.

No formal power calculations have been performed. The sample size is based on the desire to obtain adequate safety, tolerability and PK data to achieve the objectives of the study whilst exposing as few subjects as possible to study medication and procedures. Each dose group will be divided so that only one subject is dosed with active substance on the first day. For subsequent subjects in the group, dosing will be performed after at least seven days with at least 15 minutes between dosing of each subject. All safety data will be reviewed by a data monitoring committee (DMC) after each dose group.

2.2 Benefit/risk and ethical assessment

As this is a first in human study, there are no data on the effect of the drug in humans and no safe predictions about side effects are possible. Due to the biological nature of the drug, there is a risk of infusion reactions as well as allergic reactions. Subjects will be closely monitored for all adverse events and dose escalation will only occur after careful review of the safety information of subjects treated in previous dose groups. The principal investigator at Clinical Trial Unit will make sure that sufficient facilities and procedures are available to handle emergency situations during the study. Clinical Trial Unit has extended experience in phase 1 and first in man studies and there are adequate procedures in place to handle unexpected adverse reactions in study subjects. Clinical Trial Unit is located in the Lund University hospital building within a few minutes from the intensive care unit and all other specialists in the university hospital.

A conservative approach has been taken in selecting the starting dose and the dose escalation scheme for this study. The NOAEL for intravenously administered IdeS in animal toxicology studies in rabbits and dogs was found to be 2 mg/kg BW for both species (HMed Doc. No. 2012-011; 2012-012). It is also shown that the dose response curve correlates very well between *in vivo* and *in vitro* experiments in rabbits (HMed Doc. No. 2012-002). It is anticipated that this correlation is strong also in humans and therefore, the *in vitro* MABEL in humans was used together with the NOAEL to set the starting dose for this study. The selected starting dose is 0.010 mg/kg BW which is ten times below the human MABEL (0.11 mg/kg BW) and 200 times below the NOAEL. Each dose group will be divided so that only one subject is dosed on the first day. For subsequent subjects in the group, dosing will be performed when safety and efficacy data up to day 7 from the first subject in the group has been evaluated. All safety data from each dose group will be reviewed by a data monitoring committee (DMC), which will decide if it’s safe to proceed to the next dose group.

Special precautions have been taken to prevent hypersensitivity reactions. Since IdeS has its origin in *Streptococcus* and streptococcal infections are common, it is expected that a significant proportion of the population has preformed antibodies against IdeS. It is presumed that individuals with preformed IdeS antibodies of both IgE and IgG type have an increased risk of allergic reactions against IdeS. Therefore, a specific *in vitro* test system (ImmunoCap) for the quantitative measurement of IgE and IgG antibodies has been developed by Thermofisher, Uppsala, Sweden. The ImmunoCap will be used to screen study subjects before inclusion and subjects which are positive for IgE antibodies or with high IgG antibody titers (>15 mg/L) will not be included in the study.

Since IdeS degrades IgG antibodies, study subjects may have an increased risk of infection. Therefore subjects will be screened for inherited immunoglobulin disorders, e.g. IgA deficit, before inclusion in the study. Subjects participating may be unknown carriers of bacteria (for example pneumococci) with an increased risk of infection due to reduction of plasma IgG.

Strictly confidential. This document contains confidential information belonging to Hansa Medical AB

Therefore, the study subjects will receive antibiotic prophylaxis (Spektramox tablets (1x1, 500 mg/125 mg) at least until total IgG levels have returned to 6.7 g/L or more[1].

There will be a physician specialised in infectious diseases available for medical advice in case of a subject showing signs of infection. Study subjects will be instructed to contact the principal investigator immediately if they have any sign of infection. The principal investigator will then contact the infection specialist for advice on how to proceed. In case of infection in a subject with low IgG serum levels, intravenous gammaglobulin may be indicated. Subjects will also be advised to avoid living under primitive conditions until IgG have returned to acceptable levels. It is anticipated that this will occur within two to three weeks after IdeS administration.

3 STUDY OBJECTIVES

3.1 Primary objective

To reach the study maximal dose (SMD) or maximum tolerated dose (MTD) and assess the safety and tolerability of IdeS following intravenous administration of single ascending doses in healthy male subjects.

3.2 Secondary objectives

To determine the following in healthy male subjects:

1. The Pharmacokinetic (PK) profile of IdeS

2. The pharmacodynamic (PD) profile of IdeS (determined by assessing the efficacy of IdeS in cleaving IgG)

3. The immunogenicity profile of IdeS

4 STUDY PLAN AND PROCEDURES

4.1 Overall study design and flow chart

This is a Phase I, single ascending dose, double blind, randomised, study conducted in healthy male subjects at a single centre. The study design allows a gradual escalation of the dose with intensive safety monitoring to ensure the safety of the subjects. Up to 36 healthy subjects aged 18 to 45 years will participate in up to six dose groups.

The starting dose will be 0.010 mg/kg with five dose escalations. Doses can be changed based on evaluation of previous dose groups.

Subsequent dose groups will be administered increasing doses until either a non-tolerated dose is reached or the maximum allowed dose has been administered. Administration of subsequent doses of IdeS will be based on review of available data from the previous dose groups.

[1] The reference interval for total IgG is 6.7-14.5 g/L at Labmedicin Skåne.

The maximum dose administered is anticipated to be 1.2 mg/kg in this study but can be adjusted based on evaluation of previous dose groups. The provisional doses are 0.010, 0.040, 0.12, 0.36, 0.80, and 1.2 mg/kg bodyweight (BW) administered as single doses.

The infusion will be given over 30 minutes for the first two subjects in each group and over 15 minutes for subsequent subjects in the group. Dosing will be staggered. Two subjects in the dose group will be dosed on the first day (1 IdeS and 1 placebo). Providing there are no serious or unexplained safety issues, as judged by the investigator the next subjects in the group (3 IdeS and 1 placebo) will be dosed after one week (day 7) with one hour between subject numbers 3, 4 and 5. There will be 15 minutes between the last two subjects in each group. Following a 28-day screening period there will be one admission period to the clinical pharmacology unit from the day before dosing start (Day –1) until discharge on day 3 followed by several post study follow up visits. Subjects may be kept longer than 3 days in the clinic if the investigator thinks it's necessary for their safety and well-being. Administration of study drug will be on day 1 with safety monitoring and serial blood samples for PK and PD evaluation throughout the admission period (see study flow charts, table 1 and 2).

After each cohort a data monitoring committee (DMC) will assess all available safety data to make a decision on the next dose level. The DMC can decide to escalate the dose as planned, reduce or increase the dose escalation, repeat the dose, reduce the dose or terminate the study. There will be a minimum of 14 days between the last dose of one dose-level and initiation of the next dose level. All subjects must have completed a particular dose level before a higher dose group can be dosed.

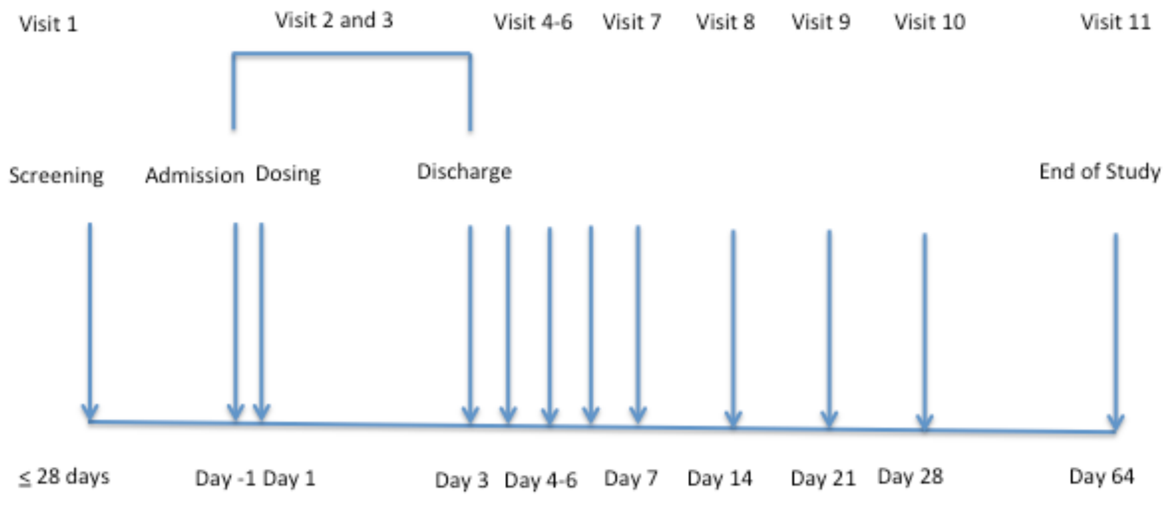

Figure 1. Study outline. Screening may be conducted over one or more days between day – 28 and day -5

Table 1A. Study flow chart for dose groups 1 and 2

Visit number	1	2	3	4	5	6	7	8	9	10	[b]*10b*	11	[d]*12*	[d]*13*
	Screening		[a]Residental									EOS		
Day	-28 to -5	-1	1-3	4	5	6	7	14	21	28	35	64	182	365
Assessment /Time window	0	0	See table 2	0	0	0	0	+/-2 d	+/-2 d	+/-2 d	+/-2 d	+/-2 d	+/-14 d	+/-14 d
Informed Consent	X													
Demographics and medical/surgical history	X													
Inclusion/exclusion	X	X												
Physical examination	X											X		
Weight	X	X						X				X		
Height	X													
[c] Vital signs (BP & pulse)	X	X	X	X	X	X	X	X	X	X		X		
[c] Body temperature	X	X	X									X		
Haematology, chemistry, coagulation, IgG and urinalysis	X	X	X	X	X	X	X	X	X	X	X	X		
Safety biomarkers	X		X											
Immunological screening	X													
Adverse events		X	X	X	X	X	X	X	X	X	X	X		
Drugs of abuse, cotinine	X	X										X		
Alcohol screen	X	X												
[c] ECG	X		X									X		
Randomisation			X											
Drug infusion			X											
PK blood sampling			X											
PD blood sampling			X											
ImmunoCap Anti-IdeS IgE and IgG	X													
ImmunoCap (ADA)			X	X			X	X	X	X		X	X	X
HIV, hepatitis B and C, syphilis and tuberculosis	X													
Concomitant medication		X	X	X	X	X	X	X	X	X	X	X		

Refer to section 7 for details. [a] Several assessments are done repeatedly during the residential period. Please refer to Table 2 for a detailed outline. [b]If IgG is still under the acceptable limit 6.7 g/L on day 28, the subject should come back for a new sample on day 35. [c] For details on vital signs, ECG and body temperature assessments please refer to sections 7.3.12 - 7.3.14. [d] Subjects will be asked to come back for anti drug antibody assessments 6 and 12 months after dosing. The results from these analyses will be reported separately. PK = Pharmacokinetic, PD = Pharmacodynamic, BP = Blood pressure.

Strictly confidential. This document contains confidential information belonging to Hansa Medical AB

Table 1B. Study flow chart for dose groups 3 to 6

Visit number	1	2	3	4	5	6	7	8	9	10	[b]*10b*	11	[d]*12*	[d]*13*
	Screening		[a]Residental									EOS		
Day	-28 to -5	-1	1-3	4	5	6	7	14	21	28	35	64	182	365
Assessment /Time window	0	0	See table 2	0	0	0	0	+/-2 d	+/-2 d	+/-2 d	+/-2 d	+/-2 d	+/-14 d	+/-14 d
Informed Consent	X													
Demographics and medical/surgical history	X													
Inclusion/exclusion	X	X												
Physical examination	X											X		
Weight	X	X						X				X		
Height	X													
[c] Vital signs (BP & pulse)	X	X	X	X	X	X	X	X	X	X		X		
[c] Body temperature	X	X	X									X		
Haematology, chemistry, coagulation, IgG and urinalysis	X	X	X	X	X	X	X	X	X	X	X	X		
Safety biomarkers	X		X											
Immunological screening	X													
Anti-tetanus and anti-CMV screening		X										X		
Adverse events		X	X	X	X	X	X	X	X	X	X	X		
Drugs of abuse, cotinine	X	X										X		
Alcohol screen	X	X												
[c] ECG	X		X									X		
Randomisation			X											
Drug infusion			X											
PK blood sampling			X	X			X							
PD blood sampling			X	X			X	X	X	X		X		
Fab/Fc blood sampling			X	X			X	X	X	X		X		
B cell receptor cleavage			X				X							
ImmunoCap Anti-IdeS IgE and IgG	X													
ImmunoCap (ADA)			X	X			X	X	X	X		X	X	X
HIV, hepatitis B and C, syphilis and tuberculosis	X													
Concomitant medication		X	X	X	X	X	X	X	X	X	X	X		

Refer to section 7 for details. [a] Several assessments are done repeatedly during the residential period. Please refer to Table 2 for a detailed outline. [b]If IgG is still under the acceptable limit 6.7 g/L on day 28, the subject should come back for a new sample on day 35. [c] For details on vital signs, ECG and body temperature assessments please refer to sections 7.3.12 - 7.3.14.

. [d] Subjects will be asked to come back for anti drug antibody assessments 6 and 12 months after dosing. The results from these analyses will be reported separately. PK = Pharmacokinetic, PD = Pharmacodynamic, BP = Blood pressure.

Table 2A. Study flow chart for residential period for dose groups 1 and 2

**Visit number**		**3 (Residental period)**								
**Day**	**1**	**1 [a]**	**1**	**1**	**1**	**1**	**1**	**1**	**2**	**3**
**Time (hours) /Assessment**	**Predose**	**0.00 min[a]**	**15 min**	**20 min**	**1**	**1 h 30 min**	**2**	**6**	**24**	**48**
**Time window (minutes)**	**≤-60 min**	**0**	**+/-1**	**+/-1**	**+/-1**	**+/-1**	**+/-1**	**+/-15**	**+/-30**	**+/-30**
**[b] Vital signs (BP& pulse)**	X		X		X		X	X		X
**[b] Body temperature**	X		X		X			X		X
**Haematology, chemistry, coagulation, IgG and urinalysis**									X	X
**Safety biomarkers**	X							(6-12h)[c]	X	X
**Adverse events**	X	X	X	X	X	X	X	X	X	X
**Drug infusion**		X								
**PK blood sampling**			14 min [d]							
**PD blood sampling**			14 min [d]							
**ImmunoCap (ADA)**	X								X	X

[a] Time 00.00 is the time point when the IdeS infusion is started. PK and PD samples should be drawn one minute before completion of the infusion. [b] For details on vital signs and body temperature assessments please refer to sections 7.3.12 - 7.3.14.. [c] Safety biomarkers at timepoint 6 hours can be drawn up to 12 hours after dosing. [d] For the first two subjects in each dose group, PK and PD samples should be drawn at 29 minutes (1 minute before end of infusion). PK = Pharmacokinetic, PD = Pharmacodynamic, BP = Blood pressure. Subjects will be monitored using telemetric ECG from predose until 24 hours after dosing.

Table 2B. Study flow chart for residential period for dose groups 3 to 6

**Visit number**		**3 (Residental period)**								
**Day**	**1**	**1 [a]**	**1**	**1**	**1**	**1**	**1**	**1**	**2**	**3**
**Time (hours) /Assessment**	**Predose**	**0.00 min[a]**	**15 min**	**20 min**	**1**	**1 h 30 min**	**2**	**6**	**24**	**48**
**Time window (minutes)**	**≤-60 min**	**0**	**+/-1**	**+/-1**	**+/-1**	**+/-1**	**+/-1**	**+/-15**	**+/-30**	**+/-30**
[b] **Vital signs (BP& pulse)**	X		X		X		X	X		X
[b] **Body temperature**	X		X		X			X		X
**Haematology, chemistry, coagulation, IgG and urinalysis**									X	X
**Safety biomarkers**	X							(6-12h)[c]	X	X
**Adverse events**	X	X	X	X	X	X	X	X	X	X
**Drug infusion**		X								
**PK blood sampling**	X		14 min [d]	X	X		X	X	X	X
**PD blood sampling**	X		14 min [d]	X	X		X	X	X	X
**Fab/Fc blood sampling**	X		14 min [d]	X	X		X	X	X	X
**B cell receptor cleavage**	X						X		X	X
**ImmunoCap (ADA)**	X								X	X

[a] Time 00.00 is the time point when the IdeS infusion is started. PK and PD samples should be drawn one minute before completion of the infusion. [b] For details on vital signs and body temperature assessments please refer to sections 7.3.12 - 7.3.14.. [c] Safety biomarkers at timepoint 6 hours can be drawn up to 12 hours after dosing. [d] For the first two subjects in each dose group. PK = Pharmacokinetic, PD = Pharmacodynamic, BP = Blood pressure. Subjects will be monitored using telemetric ECG from predose until 24 hours after dosing.

4.1.1 Stopping criteria for dose escalation

A data monitoring committee (DMC) will review available safety and tolerability data after each dose group before proceeding to the next dose. Data obtained after each dose group will be unblinded when presented to the DMC. Safety data collected up to and including the day 7 visit for the latest dose group and all available safety data from previous dose groups will be evaluated. If clinically significant changes are demonstrated in more than 2 subjects at any dose level, dose escalation will be stopped or adjusted. A well tolerated lower dose may be repeated or the same or intermediate dose may be given in another group of subjects, if considered safe by the DMC. For example, if 0 of 2 subjects on placebo and 1 of 4 subjects on active treatment are affected, dose escalation will likely continue. If 0 of 2 subjects on placebo and 2 of 4 subjects on active treatment are affected, stopping dose escalation will be considered. If dose escalation is stopped due to toxicity, the dose level below the toxic dose is considered the MTD.

The data monitoring committee will evaluate total IgG (remaining IgG in serum), as part

of the safety data, in all subjects in each group before a decision on dose escalation is made.

If unexpectedly, full effect is achieved in all subjects in one of the lower dose groups, dose escalation will not proceed to the highest group 1.2 mg/kg. Instead, dose escalation will be stopped and all PK and other data will be analysed before a decision can be made on if and how to proceed.

If full effect is achieved in a higher dose group, 0.36 or 0.80 mg/kg BW, and there are no undesired side effects, dosing will proceed to the next dose level in order to reach a dose which is expected to have a clinically relevant effect in most future patients.

4.1.2 Definition of dose limiting toxicity (DLT)

A significant reduction of total IgG levels in plasma is a desired effect of IdeS treatment and is not considered a DLT. A DLT is any other clinically relevant changes and significant changes in the safety parameters (ECG, blood pressure, body temperature, pulse, laboratory assessments and AEs) making the continuation of dosing unjustified.

4.1.3 Data Monitoring Committee (DMC)

Safety and tolerability will be evaluated after each dose level by a DMC which will be unblinded. The DMC will consist of three medical experts. The chairman of the committee will be the study team physician. The other experts will be the principal investigator and an additional physician, specialised in infectious diseases. Other relevant expertise will be consulted if deemed necessary. The members of the committee will review interim study data and decide upon the progression of dosing from one dose level of Ides to the next. Safety and tolerability data (ECG, blood pressure, body temperature, pulse, laboratory assessments and AEs) from the preceding dose group will be evaluated prior to progression to the next dose group. Data obtained more than 14 days after the dosing (AE:s, laboratory safety variables) must be review by the investigator and communicated to the DMC prior to each DMC meeting. The DMC can decide to:

- Continue to next planned dose
- Adjust the next dose level
- Postpone the next dose level
- Terminate the study

The decision of the DMC must be taken in consensus. If consensus cannot be reached, the principal investigator, who has the ultimate responsibility for the safety of the subjects, will make the final decision on the next dose level or whether to stop the study. The decisions and decision-making will be noted by the chairman and a written recommendation will be provided to the study site and Hansa Medical prior to the next scheduled dose escalation. The

DMC will have the full authority to terminate the study at any time during the course of the study.

5 SUBJECT SELECTION CRITERIA

Subject population should be selected without bias.

Investigator(s) must create and maintain a subject screening log which is a list of subjects who entered pre-trial screening but were never enrolled. Each subject must meet all of the inclusion criteria and none of the exclusion criteria for this study. Under no circumstances can there be exceptions to this rule.

5.1 Inclusion criteria

For inclusion in the study subjects must fulfill the following criteria.

1. Ability to understand and willing to sign an informed consent form.
2. Healthy male subjects aged 18 – 45 years with suitable veins for cannulation or repeated vein puncture
3. Have a body mass index (BMI) between 19 and 30 kg/m^2 and weigh at least 50 kg and no more than 100 kg

5.2 Exclusion criteria

Subjects must not enter the study if any of the following exclusion criteria are fulfilled

1. History of or diagnosis at screening of any clinically significant immunodeficiency including but not limited to immunoglobulin A deficiency
2. Tested positive for IgE antibodies against IdeS or has elevated levels of IgG anti-IdeS antibodies (>15 mg/L)* as measured by ImmunoCap.
3. History of any clinically significant disease or disorder which, in the opinion of the investigator, may either put the subject at risk because of participation in the study, or influence the results or the subject's ability to participate in the study
4. Any clinically significant illness, medical/surgical procedure or trauma within 4 weeks of the first administration of investigational product
5. Any clinically significant abnormalities in clinical chemistry, haematology, coagulation or urinalysis results as judged by the investigator

*The cut-off for anti-IdeS IgG is set to 15 mg/L, which represents the 80% percentile in an investigated population of 130 individuals. From clinical use of other bacterial proteins (e.g. Streptokinase) it is generally accepted that high levels of antibodies can neutralize the effect of the drug. The potential risk of having significant variations in exposure to pharmacologically active IdeS is the main argument for excluding subjects with high levels of anti-IdeS IgG in the first clinical study.

6. Any positive result on screening for serum hepatitis B surface antigen, hepatitis C antibody and human immunodeficiency virus (HIV), ongoing tuberculosis, ongoing syphilis, active herpes simplex or herpes zoster infection
7. After 10 minutes supine rest abnormal vital signs defined as any of the following:
   - Systolic blood pressure >140 mm Hg
   - Diastolic blood pressure > 90 mm Hg
   - Heart rate < 40 or > 100 beats per minute
8. Any clinically important abnormalities in rhythm of resting ECG as judged by the investigator
9. Known or suspected history of significant drug abuse
10. Current smokers, those who have smoked more than 20 cigarettes within the previous 6 months. Regular users of nicotine products within the previous 6 months.
11. History of alcohol abuse or excessive intake of alcohol defined as regular weekly intake of greater than 15 units of alcohol in men. 1 unit = 25 mL spirits, 125 mL wine, 250 mL beer or lager
12. Positive screen for drugs of abuse or cotinine (nicotine) at screening or on admission to the unit or positive screen for alcohol on admission to the unit prior to the first administration of investigational product
13. History of severe allergy/hypersensitivity or ongoing allergy/hypersensitivity, as judged by the investigator or history of hypersensitivity to drugs with a similar chemical structure or class to IdeS
14. Excessive intake of caffeine containing energy drinks
15. Use of any prescribed or non-prescribed medication including antacids, analgesics, herbal remedies, vitamins and minerals within two weeks prior to the first administration of investigational product and throughout the residential period
16. Plasma donation within one month of screening or any blood donation/blood loss > 500 mL during the 3 months prior to screening
17. Has received another new chemical entity (defined as a compound which has not been approved for marketing) or has participated in any other clinical study that included drug treatment within 4 months of the first administration of investigational product in this study. Subjects consented and screened but not dosed in previous phase I studies are not excluded
18. Subjects that doesn’t agree to avoid living under primitive conditions until immunoglobulin levels have returned to adequate levels as confirmed by total IgG testing and judged by the investigator
19. Previous inclusion in the present study

20. Involvement in the planning and/or conduct of the study
21. Investigator considers subject unlikely to comply with study procedures, restrictions and requirements

5.3 Withdrawal of subjects

5.3.1 Criteria for discontinuation from the study

Subjects may be discontinued from study treatment and assessments at any time. Specific reasons for discontinuation can be:

- Voluntary discontinuation by the subject who is at any time free to discontinue his participation in the study, without prejudice to further treatment
- Risk to subjects as judged by the investigator and/or sponsor
- Severe non-compliance to protocol as judged by the investigator and/or sponsor
- Incorrectly enrolled subjects or patients
- Subject lost to follow-up
- Adverse Events
- Withdrawal of informed consent to the use of biological samples may be a reason for withdrawal of a subject

Subjects who are withdrawn from the study by the investigator due to adverse events will not be replaced. Subjects who withdraw for reasons other than adverse events may be replaced.

5.3.2 Procedures for discontinuation of a subject from the study

A subject who discontinues will always be asked about the reason(s) for discontinuation and the presence of any adverse events. If possible, they will be seen and assessed by an investigator(s). Adverse events will be followed. If possible, subjects who discontinue from the study before completion should undergo the assessments and procedures scheduled for the follow-up visit.

6 STUDY CONDUCT

6.1 Restrictions during the study

The following restrictions apply from the specified time and throughout the residential period:

1. Abstain from consuming any of the following from the time specified prior to admission to the unit until and during the residential period:

- Energy drinks containing taurine or glucuronolactone e.g. Red Bull from 72 hours before admission
- Alcohol from 72 hours before admission

2. Abstain from taking any medication (prescribed or over the counter products) from 2 weeks prior to the first administration of investigational product until after the final medical examination at the study follow-up. However, paracetamol is allowed. If any other medication is necessary during the first 28 days from dosing, it should be prescribed by the investigator
3. Subjects should refrain from strenuous physical activity, which is not within the subject's normal daily routine, from 5 days prior to admission to the unit and during the residential period
4. Subjects should refrain from living under primitive conditions until IgG serum levels have returned to adequate levels as deemed by the investigator
5. Abstain from blood or plasma donation until 3 months after the final medical examination at the study follow-up

6.2 Subject enrolment

The principal investigator will:

1. Obtain signed informed consent from the potential subject before any study specific procedures are performed
2. Determine subject eligibility
3. Assign potential subject a unique subject number. If a subject or patient discontinues participation in the study, then his subject number cannot be reused

If subjects have discontinued their participation in the study then they cannot re-enter into the study.

6.3 Study treatment

6.3.1 IdeS

IdeS is a clear colourless liquid. It is formulated at 10 mg/mL in PBS and intended for intravenous administration after dilution.

Table 3. Study drug

Ingredient	Quantity	Function
IdeS drug product	10 g/L	Active ingredient
Phosphate buffered saline:		
KCl	0.20 g/L	
KH_2PO_4	0.20 g/L	Buffer
NaCl	8.0 g/L	
Na_2HPO_4	1.43 g/L	

The study will be randomised and double blinded. The hospital pharmacy will be supplied with a randomisation list produced by the CRO. The list will specify the treatment (IdeS or placebo) per subject number. IdeS will be supplied to the hospital pharmacy by Biovian in 7 mL vials packed into cartons containing 10 vials each. Vials should be kept dark at – 70°C. IdeS infusion solution or placebo infusion solution will be prepared by the hospital pharmacy and delivered in an infusion syringe including a filter containing infusion set to the clinical pharmacology unit. For subjects assigned to placebo, the pharmacy will prepare an infusion syringe with dilution buffer only (phosphate buffered saline as specified in table 3). Administration will be performed using a syringe pump. Details on ordering, preparation, labelling and administration of IdeS are described in the pharmacy manual. Code envelopes, produced by the CRO, for breaking the code in an emergency situation will be kept at the clinical pharmacology unit.

6.3.2 Doses and treatment regimens

Each subject will receive a single intravenous dose of IdeS or placebo given over 15 minutes, except for the very first subjects in dose group 1 who will receive the dose over 30 minutes. Dosing will be staggered. Two subjects in the dose group will be dosed on the first day (1 IdeS and 1 placebo). Providing there are no serious or unexplained safety issues, as judged by the investigator the next subjects in the group (3 IdeS and 1 placebo) will be dosed after one week (day 7) with 60 minutes between the first two subjects and then 15 minutes between the following.

Table 4. Dose groups. These are the anticipated dose groups but the dose may change upon evaluation of previous dose groups.

**Dose group**	**Dose**	**No of healthy subjects**	
		IdeS	Placebo
1	0.010 mg/kg BW	4	2
2	0.040 mg/kg BW	4	2
3	0.12 mg/kg BW	4	2
4	0.36 mg/kg BW	4	2
5	0.80 mg/kg BW	4	2
6	1.2 mg/kg BW	4	2

6.4 Concomitant and post-study treatment(s)

6.4.1 Prophylactic antibiotics

All subjects participating in the study will be treated with Spektramox tablets 1x1, 500 mg/125 mg (or other alternative if hypersensitive to beta-lactams) as prophylaxia against bacterial infections. Treatment will start on the dosing day and continue until day 21 or until total IgG levels are 6.7 g/L or more[1]. If total IgG levels are 6.7 g/L or more before day 21, Spektramox treatment can be stopped. Spektramox will be labeled as a non investigational medicinal product by the hospital pharmacy.

6.4.2 Other concomitant medication

No concomitant medication or therapy will be allowed except paracetamol for what is specified in the protocol during the first 28 days following dosing. The subjects must be instructed that no other medication except paracetamol is allowed including herbal remedies, vitamin supplements and over-the-counter products without the consent of the investigator.

Medication, which is considered necessary for the subject's safety and well-being, may be given at the discretion of the investigator during the residential period. When any medication is required, it should be prescribed by the study investigator. Following consultation with the sponsor, the investigator should determine whether or not the subject should continue in the study.

6.5 Treatment compliance

The administration of all medication (including investigational products) must be recorded in the appropriate sections of the CRF. Treatment compliance will be assured by supervised administration of the investigational product by the investigator or delegate. The dose, date and time of administration of the investigational product will be checked by the monitor at monitoring visits.

6.5.1 Accountability

It is the principal investigators/institution's responsibility to establish a system for handling study treatments, including investigational medicinal products, to ensure that:

1. Deliveries are correctly received by a responsible person (e.g. pharmacist or designated study personnel)

2. Deliveries are recorded

3. Study treatments are handled and stored safely and properly

4. The study drug provided for this study will be used only as directed in the study protocol

[1] The reference interval for total IgG is 6.7-14.5 g/L at Labmedicin Skåne.

5. The study personnel will account for all drugs dispensed and returned. Any discrepancies must be documented, investigated and appropriately resolved

6. At the end of the study, the pharmacy personnel will account for all unused drugs and for appropriate destruction/return of all unused drugs to the sponsor for destruction. Certificates of delivery, destruction and return must be signed by the pharmacist.

7 STUDY ASSESSMENTS

7.1 Recording of data

The investigator will ensure that all data collected in the study are provided to sponsor. He/she ensures the accuracy, completeness, legibility and timeliness of the data recorded in the appropriate sections of the paper CRF and according to any instructions provided. The principal investigator will provide sponsor with all data produced during the study from the scheduled study assessments. He/she ensures the accuracy, completeness, legibility, and timeliness of the data reported to sponsor in the CRF and in all required reports. The study assessments are described in the sections below and the timing of these assessments are detailed in the study flow charts (table 1 and 2). It is important that PK sampling occurs as close as possible to scheduled time. In order to achieve this, other assessments scheduled at the same time may be initiated prior to the time point. The timing priority order at a particular time point is:

1. Blood samples for PK and PD

2. Vital signs

3. Safety laboratory samples

4. Exploratory biomarker samples

Predose assessments may be performed up to 60 minutes prior to dosing.

7.2 Screening and demography procedures

Each subject will undergo a screening in the 28 days prior to admission. This will consist of:

1. Obtaining written informed consent prior to starting any study specific procedures are performed

2. Recording demographic data – date of birth, sex, race

3. A standard medical, medication and surgical history with review of the inclusion and exclusion criteria with the subject

4. A complete physical examination and body temperature measure

5. Height, weight and calculation of BMI

6. Vital signs – resting supine blood pressure (BP), pulse

7. Recording a resting 12-lead ECG

8. A blood sample for routine clinical chemistry, haematology, coagulation, total IgG and screen for hepatitis B surface antigen, antibodies to hepatitis C virus and antibodies to HIV

9. A blood sample for assessment of the subject's immunological profile

10. Blood samples for anti-IdeS ImmunoCap analysis and safety biomarkers

11. A urine sample for routine urinalysis, drugs of abuse screen and cotinine

12. The subject will be screened for alcohol

Subjects which are screened and eligible for inclusion may be used as back up patients. If such subject is not dosed and the 28 screening period is exceeded, the subject can be re-screened, using a simplified procedure. If the subject is still eligible, he can be used for a later dose group. The simplified re-screening must be done within 48 days from the original screening day. Re-screening will include all screening activities except the following:

- Physical examination
- ECG and vital signs
- Height
- Immunological screening
- HIV, hepatitis B and C, syphilis and tuberculosis testing

Demographics and medical/surgical history will be updated at re-screening.

7.2.1 Follow-up procedures

A post-study medical examination will be performed 64 days after the last dose. This will be similar to the one performed at screening and will include a complete physical examination, measurement of weight, vital signs, recording a 12-lead ECG, samples for clinical chemistry, haematology, coagulation, total IgG, anti-tetanus and anti-CMV, a urine sample for urinalysis, and assessment of any adverse events or required medication.

7.3 Safety

It is of the utmost importance that all staff involved in the study is familiar with the content of this section. The principal investigator is responsible for ensuring this.

7.3.1 Definition of adverse events

An adverse event (AE) is the development of an undesirable medical condition or the deterioration of a pre-existing medical condition following or during exposure to a pharmaceutical product, whether or not considered causally related to the product. Relationship to the study drug will be deemed as possible, probable or unlikely. An undesirable medical condition can be symptoms (e.g. nausea and chest pain), signs (e.g. tachycardia and enlarged liver) or the abnormal results of an investigation (e.g. laboratory findings and electrocardiogram). In clinical studies, an AE can include an undesirable medical condition occurring at any time, including run-in or washout periods, even if no study treatment has been administered.

The term AE is used to include both serious and non-serious AEs.

7.3.2 Definitions of serious adverse event

A serious adverse event is an AE occurring during any study phase (i.e. run-in, treatment, washout and follow-up), that fulfills one or more of the following criteria:

- Results in death
- Is immediately life-threatening
- Requires in-patient hospitalisation or prolongation of existing hospitalisation
- Results in persistent or significant disability or incapacity
- Is a congenital abnormality or birth defect
- Is an important medical event that may jeopardise the subject or may require medical intervention to prevent one of the outcomes listed above

7.3.3 Recording of adverse events

AEs will be collected from the time of admission to Clinical Trial Unit and throughout the study period including the follow-up period.

7.3.4 Variables

The following variables will be recorded in the CRF for each AE; description of the AE, the date and time when the AE started and stopped, Common Toxicity Criteria grade (according to CTCAE v.4.0, see Appendix 2) whether the AE is serious or not, causality rating (yes or no), action taken with regard to investigational product, if AE caused subject or patient to discontinue the study and outcome.

The Investigator will assess causal relationship between investigational product and adverse events, and answer “yes” or “no” to the question “Do you consider that there is a reasonable possibility that the event may have been caused by the investigational product.

For SAEs causal relationship will also be assessed for other medication and/or study procedure. Note that for SAEs that could be associated with any study procedure the causal relationship is implied as "yes".

7.3.5 Adverse Events based on signs and symptoms

All AEs spontaneously reported by the subject or reported in response to the open question from the study personnel: *Have you had any health problems since you were last asked?* or revealed by observation will be collected and recorded in the CRF. When collecting AEs, the recording of diagnoses is preferred (when possible) to recording a list of signs and symptoms. However, if a diagnosis is known and there are other signs or symptoms that are not generally part of the diagnosis, the diagnosis and each sign or symptom will be recorded separately.

7.3.6 Adverse Events based on examinations and tests

Deterioration as compared to baseline in protocol-mandated laboratory values, vital signs and other safety variables will only be reported as AEs if they fulfil any of the SAE criteria or are the reason for discontinuation of treatment with the investigational product. If deterioration in a laboratory value/vital sign is associated with clinical signs and symptoms, the sign/symptom will be reported as an AE and the associated laboratory result/vital sign will be considered as additional information. Any new or aggravated clinically relevant abnormal medical finding at a physical examination as compared with the baseline assessment will be reported as an AE.

Clinically relevant deterioration in non-protocol-mandated measurements will be reported as AE(s). Wherever possible the reporting investigator uses the clinical, rather than the laboratory term (e.g. anaemia *versus* low haemoglobin value).

7.3.7 Follow-up of unresolved adverse events

Any AEs that are unresolved at the patient's last AE assessment in the study are followed up by the investigator for as long as medically indicated, but without further recording in the CRF. Sponsor retains the right to request additional information for any patient with ongoing AE(s)/SAE(s) at the end of the study, if judged necessary.

7.3.8 Reporting of serious adverse events

All SAEs have to be reported, whether or not considered causally related to the investigational product, or to the study procedure(s). All SAEs will be recorded in the CRF. SAEs will be recorded from the time of admission to the clinical pharmacology unit on day -1.

The investigator and/or sponsor are responsible for informing the ethical review board and/or the regulatory authority of the SAE as per local requirements. For studies in countries implementing the EU Clinical Trials Directive, informing ethical review boards will be performed by the principal investigator. Informing regulatory authorities will be performed by the monitoring CRO on behalf of sponsor. If any SAE occurs in the course of the study, then investigators or other site personnel must inform the CRO monitor immediately but no later than the end of the next business day of when he or she becomes aware of it.

Strictly confidential. This document contains confidential information belonging to Hansa Medical AB

For fatal or life-threatening adverse events where important or relevant information is missing, active follow-up is undertaken immediately. Investigators or other site personnel inform the CRO monitor of any follow-up information on a previously reported SAE immediately but no later than the end of the next **business day** of when he or she becomes aware of it. The monitor or sponsor will advise the Investigator/study site personnel how to proceed.

Investigators or other site personnel must send relevant CRF modules by fax or e-mail to the monitoring CRO.

A Serious Adverse Event Report Form must be completed by the Investigators or other site personnel. The completed form and any other relevant supporting documentation (e.g. ECG, laboratory results and autopsy report) and relevant CRF modules should be faxed to the CRO on the same day.

The monitor works with the investigator to ensure that all the necessary information is provided to the clinical patient safety data entry site within **one business day** for fatal and life threatening events and within **five calendar** days for other SAEs. If the report arrives late in the day, it can be sent the following morning. If the report arrives during a weekend or public holiday, the information is forwarded as early as possible on the first business day following the weekend or holiday. The clock start date is then the next business day.

7.3.9 Reporting of suspected unexpected serious adverse events (SUSARs)

The sponsor is responsible for reporting suspected unexpected serious adverse events (SUSARs) to the regulatory authorities. SUSARs will be entered into the Eudravigilance Clinical Trials Module by the monitoring CRO on behalf of the sponsor. SUSARs that are fatal or life-threatening must be reported as soon as possible and in any case no later than seven days after knowledge by the sponsor. All other SUSARs must be reported as soon as possible but within a maximum of 15 days.

7.3.10 Laboratory safety assessment

Clinical chemistry, haematology, coagulation, total IgG and urine analysis

Blood samples for determination of clinical chemistry, haematology, coagulation, total IgG in plasma and urinalysis, are listed in table 5 and will be taken at the times indicated in the study flow charts (tables 1 and 2). The date and time of collection will be recorded in the CRF.

Immunological screening

An immunological screening will be performed as part of the screening procedure. This includes analysis of immunoglobulin serum levels including subclasses (IgG and subclasses, IgA, IgM) cellular immunity (CD3, CD4, CD8 and CD19 markers), levels of white blood cells (including differential count) and test of complement function, (C_3, C_4 and C-function).

Safety biomarkers

Blood samples for determination of safety biomarkers IL-6, IL-8 and TNF (approximately 5 mL) will be taken at the times indicated in the study flow charts (tables 1 and 2).

ImmunoCap anti-IdeS IgE and anti IdeS IgG analysis

A blood sample (approximately 3 mL) for detection of anti-IdeS antibodies of IgE and IgG type will be taken and analysed as part of the screening procedure. The sample will be sent to a laboratory appointed by Phadia for ImmunoCap analysis as specified in the laboratory manual.

Table 5. The following safety laboratory variables will be measured

**Clinical chemistry**	**Haematology**
P-ALT	B-Haemoglobin
P-AST	B-Leukocyte
P-Bilirubin, total	B-Absolute leukocyte differential count
P-Creatinine	B-Platelet count
P-Glucose	**Urinalysis**
P-Potassium	Urine (U)-Glucose
P-Sodium	U-Haemoglobin
P-CRP	U-Protein
P-Albumin	
**Coagulation**	**Immunological screening and total IgG**
P-APTt	S-Immunoglobulins including subclasses
P-PK (INR)	Cellular immunity (CD3, CD4, CD8 and CD19)
**Safety biomarkers**	Complement function (C_3, C_4 and C-function)
TNF	P-IgG
IL-6	**Additional safety variable**
IL-8	ImmunoCap anti-IdeS IgE
	ImmunoCap anti-IdeS IgG

Additionally, at screening all subjects will be tested for HIV, latent tuberculosis, syphilis, hepatitis B surface antigen and antibodies to hepatitis C. Urine will be tested for the following drugs of abuse at screening, admission and end of study: amphetamine, barbiturates, benzodiazepines, cocaine, buprenorphine, marijuana, methadone, methamphetamine, morphine, opiate, propoxyphene, tramadol and a separate test for cotinine. At screening and on admission the subject will be screened for alcohol. If a subject tests positive to any of these screening tests they will be excluded from the study. Laboratory values outside the reference limit suspected to be of any clinical significance will be repeated. Subjects in which suspected clinical significance is confirmed will either not be included or if already included will be followed until normalization or for as long as the investigator considers necessary. Additional laboratory variables may be performed for safety reasons if judged appropriate by the investigator. Samples for clinical chemistry, haematology, coagulation, safety biomarkers,

Strictly confidential. This document contains confidential information belonging to Hansa Medical AB

total IgG and immunological screening will be analysed using routine methods at Labmedicin Skåne. Urinalysis will be assessed by dipstick and performed by the site personnel.

7.3.11 Physical examination

For timing of individual examinations refer to the study flow charts in table 1 and 2. A complete physical examination will be performed at screening and day 64 and include an assessment of the following: general appearance, head and neck, lymph nodes, abdomen, musculo-skeletal, cardiovascular, respiratory and gross neurological examination. The investigator will pay extra attention on the presence of ongoing active infections, such as herpes simplex and herpes zoster infections. Subjects with active infections will not be included in the study.

7.3.12 Height and weight

Measurements should be taken without shoes. Body mass index will be calculated from the height and weight. The timing of individual measurements is indicated in the study flow charts in table 1 and 2.

7.3.13 ECG

ECG 12-lead /supine position will be taken after 10 minutes supine rest at screening, and on day 64. At dosing, subjects will be monitored using telemetric ECG from predose until 24 hours after dosing.

7.3.14 Vital signs

Pulse and blood pressure:
Supine blood pressure and pulse will be measured at screening and at all regular visits. At dosing, supine blood pressure and pulse will be recorded pre-dose, 15 minutes and 1, 2, 6 and 48 hours post-dose. In addition, the subjects will be monitored with a 5 lead telemetry during the infusion and the following 24 hours.

Other safety assessments:
Body temperature will be measured as specified in the study flow charts (tables 1 and 2). At dosing, body temperature will be recorded pre-dose, 15 min, 1, 6 and 48 hours post-dose.

7.4 Pharmacokinetics and Pharmacodynamics

7.4.1 Collection of biological samples

Venous blood samples (approximately 5 mL) for the determination of concentrations of IdeS (PK) in serum will be taken at the times presented in the study flow charts (table 1 and 2). The date and time of collection of each sample will be recorded on the laboratory requisition form and in the CRF. Venous blood samples (approximately 5 mL) for the determination of IgG levels in serum (PD) will be taken at the times presented in the study flow charts (table 1 and 2). The date and actual time of collection of each sample will be recorded on the laboratory requisition form and in the CRF. Samples will be collected, labelled stored and shipped as detailed in the laboratory manual. For subjects in dose group 1 and 2, only one PK and one PD sample will be drawn as indicated in table 2A.

7.4.2 Determination of drug concentration in biological samples

Samples for determination of IdeS concentration in serum (PK) will be analysed by Covance analysis laboratory, Harrogate, England. Full details of the analytical method used will be detailed in a separate bioanalytical report.

7.4.3 Determination of IgG levels in serum

Samples for determination of IgG levels in serum (PD) will be analysed by Covance analysis laboratory, Harrogate, England. Full details of the analytical method used will be detailed in a separate bioanalytical report.

7.5 Anti-drug antibody analysis

7.5.1 Collection of biological samples

Venous blood samples (approximately 3 mL) for the determination of anti drug antibody (ADA) levels in serum will be taken at the times presented in the study flow charts (table 1 and 2). The date and time of collection of each sample will be recorded on the laboratory requisition form and in the CRF. Samples will be collected, labelled stored and shipped as detailed in the laboratory manual.

7.5.2 Determination of ADA levels in serum

Determination of IgG anti-IdeS antibody concentrations in serum will be performed using anti-IdeS ImmunoCap, developed by Phadia, Uppsala, Sweden . Full details of the analytical method used will be detailed in a separate bioanalytical report.

7.6 Exploratory biomarker analysis

7.6.1 Determination of anti-tetanus and anti-CMV levels in serum

Venous blood samples (approximately 5 mL) for the determination of anti-tetanus and anti-CMV antibody levels in serum will be taken at the times presented in the study flow chart (table 1). The date and time of collection of each sample will be recorded on the laboratory requisition form and in the CRF. Samples for anti-tetanus and anti-CMV will be analysed using routine methods at Labmedicin Skåne. There will be no anti-tetanus or anti-CMV analysis for subjects in dose group 1 and 2.

7.6.2 Determination of Fc and Fab in serum

Venous blood samples (approximately 3 mL) for the determination of Fab2/Fc fragment analysis in serum will be taken at the times specified in the study flow chart (table 1). The date and time of collection of each sample will be recorded on the laboratory requisition form and in the CRF. The kinetics of the Fab and Fc fragments generated by IdeS cleaving IgG will be measured by Hansa Medical, Lund using a research laboratory method.. There will be no Fab/Fc fragment analysis for subjects in dose group 1 and 2.

7.6.3 Detection of B cell receptor cleavage *in vivo*

Venous blood samples (approximately 5 mL) for analysis of B cell receptor cleavage in vivo in plasma will be taken at the times specified in the study flow charts (tables 1B and 2B). The

date and time of collection of each sample will be recorded on a separate form. The B cell receptor cleavage will be measured by Hansa Medical, Lund using a research laboratory method. There will be no B cell receptor analysis for subjects in dose group 1 and 2.

8 BIOLOGICAL SAMPLING PROCEDURES

8.1 Volume of blood

The maximum volume to be drawn from each subject or patient in the study will not exceed 450 mL, i.e. the same volume as would be drawn during a regular blood donation.

8.2 Handling, storage and destruction of biological samples

The samples will be used or disposed after analyses.

8.2.1 Pharmacokinetic, pharmacodynamic and anti-drug antibody samples

Samples will be disposed after the clinical study report has been finalised, unless retained for future analyses, see below.

Key samples for validation may be retained at the analysing CRO laboratory on behalf of sponsor for a maximum of one year following the finalisation of the clinical study report. The results from the validation will not be reported in the Clinical Study Report but separately in the bioanalytical method validation report.

8.3 Chain of custody of biological samples

A full chain of custody is maintained for all samples throughout their lifecycle.

The principal investigator keeps full traceability of collected biological samples from the subjects while in storage at the centre until shipment and keeps documentation of receipt of arrival.

The sample receiver keeps full traceability of the samples while in storage and during use until used or disposed.

Sponsor keeps oversight of the entire life cycle through internal procedures, monitoring of study sites and auditing of external laboratory providers.

8.4 Withdrawal of informed consent for donated biological samples

If a subject or patient withdraws consent to the use of biological samples donated the samples will be disposed/destroyed, if not already analysed and documented.

The principal investigator:

- Ensures subject or patient withdrawal of informed consent is notified immediately to sponsor

- Ensures that biological samples from that subject or patient, if stored at the study site, are immediately identified, disposed/destructed and the action documented.

- Ensures the laboratory(ies) holding the samples is/are informed about the withdrawn consent immediately and that samples are disposed/destructed and the action documented returned to the study site.

Sponsor ensures the laboratory(ies) holding the samples is/are informed about the withdrawn consent immediately and that samples are disposed/destructed and the action documented returned to the study site.

In the event that analysis/research has already been performed, sponsor will retain the results and associated data for regulatory reasons but these will not be used in any subsequent analyses.

9 ETHICAL AND REGULATORY REQUIREMENTS

9.1 Ethical conduct of the study

The study will be performed in accordance with ethical principles that have their origin in the Declaration of Helsinki and are consistent with ICH/Good Clinical Practice, applicable regulatory requirements.

9.2 Subject data protection

The informed consent form (ICF) will incorporate wording that complies with relevant data protection and privacy legislation.

9.3 Ethics and regulatory review

An ethical review board must approve the final study protocol, including the final version of the ICF and any other written information to be provided to the subjects. The investigator will ensure the distribution of these documents to the applicable ethical review board, and to the study site staff. The opinion of the ethical review board must be given in writing.

The ethical review board must approve all advertising used to recruit subjects for the study.

Sponsor must approve any modifications to the ICF that are needed to meet local requirements.

Before enrolment of any subject or patient into the study, the final study protocol, and the final version of the informed consent form are approved by the national regulatory authority or a notification to the national regulatory authority is done, according to local regulations.

The distribution of any of these documents to the national regulatory authorities will be handled by sponsor.

Sponsor will provide ethical review boards and principal investigators with safety updates/reports according to local requirements.

Strictly confidential. This document contains confidential information belonging to Hansa Medical AB

Progress reports and notifications of serious and unexpected adverse drug reactions will be provided to the ethical review board according to local regulations and guidelines.

9.4 Informed consent

The principal investigator(s) at the centre will:

- Ensure that the subject or patient is given full and adequate oral and written information about the nature, purpose, possible risk and benefit of the study.
- Ensure that the subjects are notified that they are free to discontinue the study at any time.
- Ensure that the subject or patient is given the opportunity to ask questions and allowed time to consider the information provided.
- Obtain and document the subject's or patient's signed and dated informed consent before conducting any procedure specifically for the study.
- Ensure the original, signed informed consent form is stored in the Investigator's Study File.
- Ensure a copy of the signed informed consent form is given to the subject or patient.

9.5 Changes to the protocol and informed consent form

Study procedures will not be changed without the mutual agreement of the principal investigator and sponsor.

If there are any substantial changes to the study protocol, then these changes will be documented in a study protocol amendment.

The amendment must be approved by the ethical review board before implementation. Local requirements must be followed for amended protocols.

Sponsor will distribute any subsequent amendments and new versions of the protocol to the principal investigator.

If a protocol amendment requires a change to a centre's informed consent form, sponsor and the centre's ethical review board must approve the revised informed consent form before the revised form is used.

If local regulations require, any administrative change will be communicated to or approved by the ethical review board.

Strictly confidential. This document contains confidential information belonging to Hansa Medical AB

9.6 Audits and inspections

Authorized representatives of sponsor or a regulatory authority may perform audits or inspections at the centre, including source data verification. The purpose of an audit or inspection is to systematically and independently examine all study-related activities and documents, to determine whether these activities were conducted, and data were recorded, analysed, and accurately reported according to the protocol, Good Clinical Practice (GCP), guidelines of the International Conference on Harmonisation (ICH), and any applicable regulatory requirements. The investigator will contact sponsor immediately if contacted by a regulatory agency about an inspection at the centre.

10 STUDY MANAGEMENT

10.1 Pre-study activities

Before the first subject is entered into the study, it is necessary for a representative of sponsor to visit the investigational study site to:

- Determine the adequacy of the facilities
- Discuss with the investigator(s) (and other personnel involved with the study) their responsibilities with regard to protocol adherence and the responsibilities of sponsor or its representatives. This will be documented in a clinical study agreement between sponsor and the investigator

10.2 Training of study site personnel

Before the first subject is entered into the study, a sponsor representative will review and discuss the requirements of the clinical study protocol and related documents with the investigational staff and also train them in any study specific procedures and system(s) utilised.

The principal investigator will ensure that appropriate training relevant to the study is given to all staff, and that any new information relevant to the performance of this study is forwarded to the staff involved.

The principal investigator will maintain a record of all individuals involved in the study (medical, nursing and other staff).

10.3 Monitoring of the study

During the study, a sponsor representative will have regular contacts with the study site, including visits to:

- Provide information and support to the investigator(s).
- Confirm that facilities remain acceptable.

- Confirm that the investigational team is adhering to the protocol, that data are being accurately and timely recorded in the CRFs, and that investigational product accountability checks are being performed.

- Perform source data verification (a comparison of the data in the CRFs with the subject's medical records at the hospital or practice, and other records relevant to the study) incl. verification of informed consent of participating subjects. This will be done using print outs of records for each subject or patient.

- Ensure withdrawal of informed consent to the use of the subject's biological samples is reported and biological samples are identified and disposed/destructed accordingly, and the action is documented, and reported to the subject.

The Sponsor and the CRO will be available between visits if the investigator(s) or other staff at the centre needs information and advice about the study conduct.

10.3.1 Source data

Refer to the source data document for location of source data.

10.4 Study agreements

The principal investigator at the centre must comply with all the terms, conditions, and obligations of the clinical study agreement for this study. In the event of any inconsistency between this clinical study protocol and the clinical study agreement, the clinical study protocol shall prevail.

Agreements between sponsor and the principal investigator must be in place before any study-related procedures can take place, or subjects be enrolled.

10.5 Study timetable and end of study

The end of the entire study is defined as "the last visit of the last subject undergoing the trial".

The study is expected to start in Quarter 1 2013 and to be completed by Quarter 4 2013.

Sponsor may terminate the entire study prematurely if concerns for safety arise within this study.

11 DATA MANAGEMENT

All data management will be performed by the data management unit at Commitum/Norma. The data management procedure will be performed in accordance with ICH guidelines and Commitum/Norma's standard operating procedures (SOP) and conducted in accordance with good clinical, scientific and data management principles.

The investigator is responsible for ensuring the accuracy, completeness, legibility and timelines of the data recorded in the case report forms (CRF's). Any changes or corrections to CRFs must be dated, initialled and explained (if necessary) by the investigator or delegated

study personnel. Signed sections of CRFs should be monitored and collected on a regular basis. Any corrections after CRF pages have been collected will be done using data clarification forms (DCF).

All the data in the CRFs will be reviewed for legibility and entered into a SAS database which will be subject to both human visual and computer driven procedures in order to maximize logical consistencies in the collected CRF data. All data will be single entered with 10% verification.

All inconsistencies detected by these procedures will be resolved by using data correction forms (DCFs). The investigator will either confirm that the CRF data is correct, or alternatively record the correct data on the DCF. A copy of the DCF will be filed with the CRF at the Investigational site and a signed copy will be returned to Commitum/Norma's.

Before the database can be declared complete and accurate a final quality control will be made. An electronically generated random sample of 10% of the subject will be 100% proofread against listings.

When the database has been declared to be complete and accurate, the database will be locked and un-blinded. Any changes to the database after that time can only be made by joint written agreement between the CRM, the data manager and the biostatistician.

The CRFs will be sent to sponsor for archiving after the statistical report is finalised.

12 EVALUATION AND CALCULATION OF VARIABLES

12.1 Calculation or derivation of pharmacokinetic variables

Pharmacokinetic analysis of the serum concentration data for IdeS will be performed at the appointed CRO on behalf of sponsor. The actual sampling times will be used in the parameter calculations and PK parameters will be derived using standard non-compartmental methods. Concentration-time raw data and pharmacokinetic parameters will be calculated for each individual as well as reported per dose group.

Where possible, the following PK parameters will be determined for IdeS: Maximum plasma concentration ($C_{max} = C_{0hr}$), Time at which maximum plasma concentration is observed (t_{max}), terminal rate constant (λ_z); terminal half-life (t½ë), area under the concentration-time curve (AUCt), Area under the concentration-time curve from time 0 to infinity (AUCinf), Area under the concentration-time curve for the last observed concentration (AUClast), Area under the serum concentration-time curve in a dosing interval (AUCtau), Percentage of AUC that is due to extrapolation from the last concentration to infinity (AUCextra%), apparent plasma clearance (CL), apparent volume of distribution during terminal phase (V_z). Volume of distribution based at steady state (Vss), and Mean Residence Time extrapolated to infinity (MRT).

12.2 Calculation or derivation of pharmacodynamic variables

12.2.1 Calculation or derivation of the relationship between pharmacokinetic and pharmacodynamic variables

Relationship between IdeS serum or plasma concentration and effect of IgG cleavage will be presented graphically. If appropriate, concentration-effect relationships will be explored.

13 STATISTICAL METHODS AND SAMPLE SIZE

13.1 Description of analysis sets

13.1.1 General principles

All data available for each dose group will be used in the analysis for safety, PK and pharmacodynamics. Prior to clean file will a detailed Statistical Analysis Plan be written, which will include a description of any data deletion and the rationale for this.

13.2 Methods of statistical analyses

13.2.1 General principles

Given the exploratory nature, no formal statistical hypothesis testing will be performed in this study. Data will be presented by actual dose received.

13.2.2 Subject characteristics

The following demographic variables will be listed: age, sex, race, height, weight and BMI for all dose groups as appropriate.

Medical/surgical histories and medications taken before screening will be listed including comments and coded fields.

Baseline assessments of safety variables (ECG, vital signs, and laboratory parameters) will be listed by dose groups as appropriate.

13.2.3 Safety and tolerability

All safety and tolerability data will be presented by dose group using listings as specified in the SAP.

Adverse events will be collected for each subject from admission (Day -1, Visit 2) until follow-up (Visit 11). Serious adverse events will be recorded from the time when informed consent is obtained (Visit 1) until the follow-up visit. AEs that occur before dosing will be reported separately.

Adverse events will be summarised by PT (Preferred term) and SOC (System organ class) using MedDRA vocabulary. Furthermore, listings of serious adverse events and adverse events that led to withdrawal will be made and the number of subjects who had any adverse

events, serious adverse events, adverse events that led to withdrawal, and adverse events with severe intensity will be summarised.

13.2.4 Pharmacokinetics

Pharmacokinetic variables will be summarised using appropriate descriptive statistics (e.g. n, geometric mean, coefficient of variance (CV), min, median, max) by treatment group. Dose linearity will be statistically evaluated.

13.2.5 Pharmacodynamics

All data associated with the efficacy of IdeS in cleaving IgG will be presented using listings for each dose group.

13.3 Determination of sample size

Due to the exploratory nature of the study the sample size is not based on formal statistical considerations. The sample size is based on experience from previous similar Phase I studies with other compounds to obtain adequate safety, tolerability and PK data to achieve the objectives of the study whilst exposing as few subjects as possible to study medication and procedures.

14 REFERENCES

2012-002 (2012). IdeS dose titration *in vitro* and calculation of Minimum Anticipated Biologically Effect Level (MABEL) and Minimum Effectice Dose (MED) in rabbit and human.

2012-003 (2012). *In vitro* IdeS efficacy in human, rabbit, dog, cynomolgus monkey, rhesus macaque and common marmoset.

2012-011 (2012). IdeS rabbit pivotal toxicity report.

2012-012 (2012). IdeS dog pivotal toxicity report.

Agniswamy J., Lei B., Musser J. M. and Sun P. D. (2004). Insight of host immune evasion mediated by two variants of group a Streptococcus Mac protein. *J Biol Chem*. **279**: 52789-96

Akalin E., Dinavahi R., Friedlander R., Ames S., de Boccardo G., Sehgal V., Schroppel B., Bhaskaran M., Lerner S., Fotino M., Murphy B. and Bromberg J. S. (2008). Addition of plasmapheresis decreases the incidence of acute antibody-mediated rejection in sensitized patients with strong donor-specific antibodies. *Clin J Am Soc Nephrol*. **3**: 1160-7

Archdeacon P., Chan M., Neuland C., Velidedeoglu E., Meyer J., Tracy L., Cavaille-Coll M., Bala S., Hernandez A. and Albrecht R. (2011). Summary of FDA antibody-mediated rejection workshop. *Am J Transplant*. **11**: 896-906

Bächler K., Amico P., Honger G., Bielmann D., Hopfer H., Mihatsch M. J., Steiger J. and Schaub S. (2010). Efficacy of induction therapy with ATG and intravenous immunoglobulins in patients with low-level donor-specific HLA-antibodies. *Am J Transplant*. **10**: 1254-62

Chinen J. and Buckley R. H. (2010). Transplantation immunology: solid organ and bone marrow. *J Allergy Clin Immunol*. **125**: S324-35

Colvin R. B. (2007). Antibody-mediated renal allograft rejection: diagnosis and pathogenesis. *J Am Soc Nephrol*. **18**: 1046-56

Colvin R. B. and Smith R. N. (2005). Antibody-mediated organ-allograft rejection. *Nat Rev Immunol*. **5**: 807-17

Gebel H. M., Bray R. A. and Nickerson P. (2003). Pre-transplant assessment of donor-reactive, HLA-specific antibodies in renal transplantation: contraindication vs. risk. *Am J Transplant*. **3**: 1488-500

Gloor J., Cosio F., Lager D. J. and Stegall M. D. (2008). The spectrum of antibody-mediated renal allograft injury: implications for treatment. *Am J Transplant*. **8**: 1367-73

Gloor J. M., DeGoey S. R., Pineda A. A., Moore S. B., Prieto M., Nyberg S. L., Larson T. S., Griffin M. D., Textor S. C., Velosa J. A., Schwab T. R., Fix L. A. and Stegall M. D. (2003). Overcoming a positive crossmatch in living-donor kidney transplantation. *Am J Transplant*. **3**: 1017-23

Higgins R. M., Bevan D. J., Carey B. S., Lea C. K., Fallon M., Buhler R., Vaughan R. W., O'Donnell P. J., Snowden S. A., Bewick M. and Hendry B. M. (1996). Prevention of hyperacute rejection by removal of antibodies to HLA immediately before renal transplantation. *Lancet*. **348**: 1208-11

Johansson B. P., Shannon O. and Björck L. (2008). IdeS: a bacterial proteolytic enzyme with therapeutic potential. *PLoS One*. **3**: e1692

Jordan S. C., Tyan D., Stablein D., McIntosh M., Rose S., Vo A., Toyoda M., Davis C., Shapiro R., Adey D., Milliner D., Graff R., Steiner R., Ciancio G., Sahney S. and Light J. (2004). Evaluation of intravenous immunoglobulin as an agent to lower allosensitization and improve transplantation in highly sensitized adult patients with end-stage renal disease: report of the NIH IG02 trial. *J Am Soc Nephrol*. **15**: 3256-62

Jordan S. C., Vo A., Bunnapradist S., Toyoda M., Peng A., Puliyanda D., Kamil E. and Tyan D. (2003). Intravenous immune globulin treatment inhibits crossmatch positivity and

allows for successful transplantation of incompatible organs in living-donor and cadaver recipients. *Transplantation*. **76**: 631-6

Jordan S. C., Vo A. A., Peng A., Toyoda M. and Tyan D. (2006). Intravenous gammaglobulin (IVIG): a novel approach to improve transplant rates and outcomes in highly HLA-sensitized patients. *Am J Transplant*. **6**: 459-66

Kissmeyer-Nielsen F., Olsen S., Petersen V. P. and Fjeldborg O. (1966). Hyperacute rejection of kidney allografts, associated with pre-existing humoral antibodies against donor cells. *Lancet*. **2**: 662-5

Lefaucheur C., Nochy D., Hill G. S., Suberbielle-Boissel C., Antoine C., Charron D. and Glotz D. (2007). Determinants of poor graft outcome in patients with antibody-mediated acute rejection. *Am J Transplant*. **7**: 832-41

Lefaucheur C., Suberbielle-Boissel C., Hill G. S., Nochy D., Andrade J., Antoine C., Gautreau C., Charron D. and Glotz D. (2008). Clinical relevance of preformed HLA donor-specific antibodies in kidney transplantation. *Am J Transplant*. **8**: 324-31

Lucas J. G., Co J. P., Nwaogwugwu U. T., Dosani I. and Sureshkumar K. K. (2011). Antibody-mediated rejection in kidney transplantation: an update. *Expert Opin Pharmacother*. **12**: 579-92

Mai M. L., Ahsan N., Wadei H. M., Genco P. V., Geiger X. J., Willingham D. L., Taner C. B., Hewitt W. R., Grewal H. P., Nguyen J. H., Hughes C. B. and Gonwa T. A. (2009). Excellent renal allograft survival in donor-specific antibody positive transplant patients-role of intravenous immunoglobulin and rabbit antithymocyte globulin. *Transplantation*. **87**: 227-32

Moll S. and Pascual M. (2005). Humoral rejection of organ allografts. *Am J Transplant*. **5**: 2611-8

Montgomery R. A., Lonze B. E., King K. E., Kraus E. S., Kucirka L. M., Locke J. E., Warren D. S., Simpkins C. E., Dagher N. N., Singer A. L., Zachary A. A. and Segev D. L. (2011). Desensitization in HLA-incompatible kidney recipients and survival. *N Engl J Med*. **365**: 318-26

Montgomery R. A. and Zachary A. A. (2004). Transplanting patients with a positive donor-specific crossmatch: a single center's perspective. *Pediatr Transplant*. **8**: 535-42

Montgomery R. A., Zachary A. A., Ratner L. E., Segev D. L., Hiller J. M., Houp J., Cooper M., Kavoussi L., Jarrett T., Burdick J., Maley W. R., Melancon J. K., Kozlowski T., Simpkins C. E., Phillips M., Desai A., Collins V., Reeb B., Kraus E., Rabb H., Leffell M. S. and Warren D. S. (2005). Clinical results from transplanting incompatible live kidney donor/recipient pairs using kidney paired donation. *JAMA*. **294**: 1655-63

Strictly confidential. This document contains confidential information belonging to Hansa Medical AB

Morath C., Beimler J., Opelz G., Scherer S., Schmidt J., Macher-Goeppinger S., Klein K., Sommerer C., Schwenger V., Zeier M. and Susal C. (2012). Living donor kidney transplantation in crossmatch-positive patients enabled by peritransplant immunoadsorption and anti-CD20 therapy. *Transpl Int*. **25**: 506-17

Nandakumar K. S., Collin M., Olsen A., Nimmerjahn F., Blom A. M., Ravetch J. V. and Holmdahl R. (2007). Endoglycosidase treatment abrogates IgG arthritogenicity: importance of IgG glycosylation in arthritis. *Eur J Immunol*. **37**: 2973-82

Ojo A. O., Wolfe R. A., Held P. J., Port F. K. and Schmouder R. L. (1997). Delayed graft function: risk factors and implications for renal allograft survival. *Transplantation*. **63**: 968-74

Patel R. and Terasaki P. I. (1969). Significance of the positive crossmatch test in kidney transplantation. *N Engl J Med*. **280**: 735-9

Reinsmoen N. L., Lai C. H., Vo A., Cao K., Ong G., Naim M., Wang Q. and Jordan S. C. (2008). Acceptable donor-specific antibody levels allowing for successful deceased and living donor kidney transplantation after desensitization therapy. *Transplantation*. **86**: 820-5

Susal C. and Morath C. (2011). Current approaches to the management of highly sensitized kidney transplant patients. *Tissue Antigens*. **77**: 177-86

Terasaki P. I. and Ozawa M. (2004). Predicting kidney graft failure by HLA antibodies: a prospective trial. *Am J Transplant*. **4**: 438-43

Wenig K., Chatwell L., von Pawel-Rammingen U., Björck L., Huber R. and Sondermann P. (2004). Structure of the streptococcal endopeptidase IdeS, a cysteine proteinase with strict specificity for IgG. *Proc Natl Acad Sci U S A*. **101**: 17371-6

Vincents B., von Pawel-Rammingen U., Björck L. and Abrahamson M. (2004). Enzymatic characterization of the streptococcal endopeptidase, IdeS, reveals that it is a cysteine protease with strict specificity for IgG cleavage due to exosite binding. *Biochemistry*. **43**: 15540-9

von Pawel-Rammingen U., Johansson B. P. and Björck L. (2002). IdeS, a novel streptococcal cysteine proteinase with unique specificity for immunoglobulin G. *EMBO J*. **21**: 1607-15
