# Supplementary figures and images for: Complete Removal of Extracellular IgG Antibodies in a Randomized Dose-Escalation Phase I Study with the Bacterial Enzyme IdeS – A Novel Therapeutic Opportunity
Source: PLoS One. 2015 Jul 15;10(7):e0132011. doi: 10.1371/journal.pone.0132011 (PMC4503742; doi:10.1371/journal.pone.0132011)

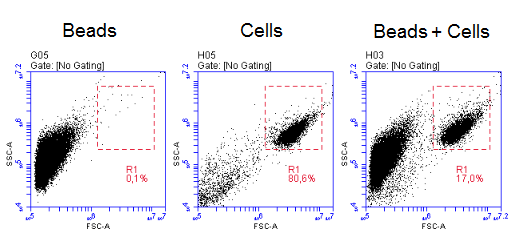

Supplement: S1 Fig — The left plot shows the forward and side scatter of the fluorescent beads. The middle plot shows the scatter for the THP-1 cells and the right plot shows the scatter for cells and beads when mixed together. Cells are clearly separated from beads and the gated area (R1) is shown in the plots. (TIF) [file pone.0132011.s004.tif]

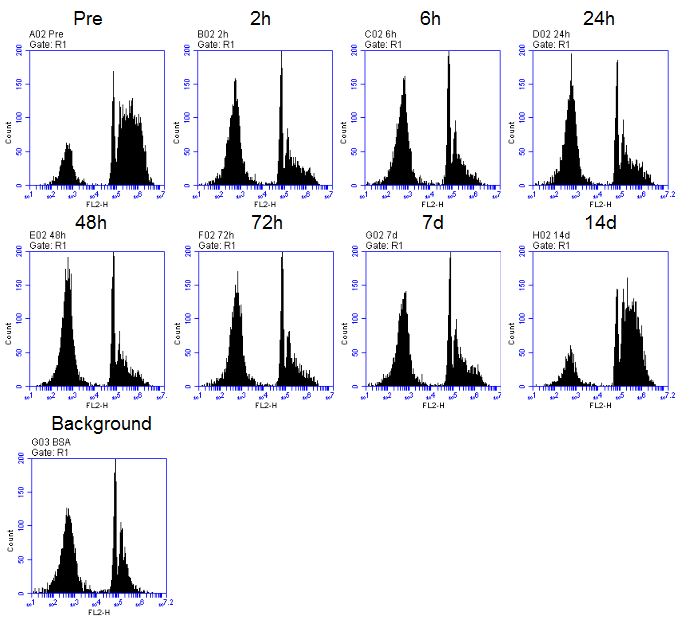

Supplement: S2 Fig — Cells were gated in R1 (see S1 Fig) and further monitored for bead uptake in FL2. The graphs show the phagocytic potential of IgG and IgG-fragments present in serum collected at different time-points post dosing of 0.24 mg/kg BW of IdeS. Above each graph is the time-point post dosing shown, with pre-dose in the upper left corner. The last plot shows background which is bead uptake in the absence of serum. (TIF) [file pone.0132011.s005.tif]
